# Metabolomics and computational analysis of the role of monoamine oxidase activity in delirium and SARS-COV-2 infection

**DOI:** 10.1038/s41598-021-90243-1

**Published:** 2021-05-20

**Authors:** Miroslava Cuperlovic-Culf, Emma L. Cunningham, Hossen Teimoorinia, Anuradha Surendra, Xiaobei Pan, Steffany A. L. Bennett, Mijin Jung, Bernadette McGuiness, Anthony Peter Passmore, David Beverland, Brian D. Green

**Affiliations:** 1 Digital Technologies Research Centre, National Research Council of Canada, Ottawa, Canada; 2 Department of Biochemistry, Microbiology, and Immunology, University of Ottawa, Ottawa, ON K1H 8M5 Canada; 3 Centre for Public Health, Queen’s University Belfast, Block B, Institute of Clinical Sciences, Royal Victoria Hospital Site, Grosvenor Road, Belfast, BT12 6BA Northern Ireland; 4 NRC Herzberg Astronomy and Astrophysics, 5071 West Saanich Road, Victoria, BC V9E 2E7 Canada; 5 Institute for Global Food Security, School of Biological Sciences, Queen’s University Belfast, 8 Malone Road, Belfast, BT9 5BN Northern Ireland; 6 Neural Regeneration Laboratory, Ottawa Institute of Systems Biology, Brain and Mind Research Institute, Department of Biochemistry, Microbiology, and Immunology, University of Ottawa, Ottawa, ON Canada; 7 Outcomes Assessment Unit, Musgrave Park Hospital, Stockman’s Lane, Belfast, BT9 7JB Northern Ireland

**Keywords:** Computational models, Data mining, Neurological disorders, Neural ageing

## Abstract

Delirium is an acute change in attention and cognition occurring in ~ 65% of severe SARS-CoV-2 cases. It is also common following surgery and an indicator of brain vulnerability and risk for the development of dementia. In this work we analyzed the underlying role of metabolism in delirium-susceptibility in the postoperative setting using metabolomic profiling of cerebrospinal fluid and blood taken from the same patients prior to planned orthopaedic surgery. Distance correlation analysis and Random Forest (RF) feature selection were used to determine changes in metabolic networks. We found significant concentration differences in several amino acids, acylcarnitines and polyamines linking delirium-prone patients to known factors in Alzheimer’s disease such as monoamine oxidase B (MAOB) protein. Subsequent computational structural comparison between MAOB and angiotensin converting enzyme 2 as well as protein–protein docking analysis showed that there potentially is strong binding of SARS-CoV-2 spike protein to MAOB. The possibility that SARS-CoV-2 influences MAOB activity leading to the observed neurological and platelet-based complications of SARS-CoV-2 infection requires further investigation.

## Introduction

COVID-19 is an ongoing major global health emergency caused by severe acute respiratory syndrome coronavirus SARS-CoV-2. Patients admitted to hospital with COVID-19 show a range of features including fever, anosmia, acute respiratory failure, kidney failure and gastrointestinal issues and the death rate of infected patients is estimated at 2.2%^1^. Some early studies of hospitalized patients indicated that 20–30% of COVID-19 patients develop some form of delirium or mental status change rising to 60–70% for those patients with severe illness of all age groups^2,3^. The exact mechanisms are not understood although a number of possible causes have been proposed including direct viral invasion, cerebrovascular involvement, hypoxia, pyrexia, dehydration, hyperinflammation (cytokine storm), medications or metabolic derangements^2,3^. The X-ray structure of SARS-CoV-2 spike protein binding to Angiotensin-converting enzyme 2 (ACE2)^4,5^ as well as recently demonstrated specific structural features of SARS-CoV-2 spike protein^6^, suggest specific features of SARS-CoV-2 spike protein structure for binding to human protein in the SARS-CoV-2 influence virulence and major relevance of this protein and its interactions in the development of drugs against the virus^7,8^.

Delirium, an acute disorder of attention and cognition^9^ is an unpleasant experience for patients, relatives and healthcare staff and is associated with negative outcomes such as dementia and death. As has been described by Fong et al.^10^, whilst even those with the most resilient of brains can develop delirium in the face of severe stressors, delirium in the face of more moderate insults may be a sign of underlying neurodegeneration^11^. An improved understanding of the causes of postoperative delirium could provide a better appreciation of the vulnerabilities causing delirium following surgery, as well as following SARS-CoV-2 infection.

Planned orthopedic surgery under spinal anaesthesia provides a unique opportunity to preoperatively sample cerebrospinal fluid (CSF) in a group of patients where an estimated 17% will subsequently develop delirium^12^. Metabolomic analysis of body fluids, including blood and CSF, provides a wide-ranging molecular window into the major processes of the body including an insight into the brain metabolism. CSF metabolite biomarkers of delirium risk have already been identified^13^. Major differences in the concentrations of polyamines (including spermidine and putrescine) are present in delirium-prone patients even before surgery or the presentation of delirium. However, it is not clear whether such metabolic changes occur more peripherally in the blood circulation, or whether the transfer of these metabolite across the blood–brain barrier is important.

Recently, Shen et al.^14^ presented metabolomics and proteomics analysis of serum of mild and severe COVID-19 patients indicating major concentration differences in serotonin, kynurenine, a number of amino acids, as well as, alterations in tryptophan and polyamine metabolic pathways. The metabolomics data were in agreement with clinical studies showing coagulopathy, stimulated by platelets, as one of the major issues in severe COVID-19 cases^14^, Additionally, delirium, possibly associated with changes in neurotransmitters has been indicated as a severe consequence of SARS-CoV-2 infection in some patients^15^. The involvement of the mitochondrial membrane bound enzyme monoamine oxidase (MAO) emerges as a common potential candidate causing many of the observed COVID-19 side-effects. MAO is important for neurotransmitter metabolism, has prior associations with delirium, and is involved in platelet regulation and coagulation^16–18^ as well as anosmia^19^. MAO including MAOA and MAOB are flavoenzymes that catalyze the oxidative transformation of monoamines. Inhibition of these enzymes is an established therapeutic target which is still in development, and the selectivity of candidate molecules for one isoform over another is a key consideration^19–21^. Alterations in the activities of MAOs is a potential source of various neuropsychiatric disorders including depression, autism or aggressive behavior^22,23^. Additionally, the function of MAOs represents an inherent source of oxidative stress, leading to the damage and death of neurons, ultimately leading to neurodegenerative diseases such as Parkinson's or Alzheimer's disease^24,25^.

## Materials and methods

### Samples and experimental analyses

Preoperative blood and CSF samples were collected within an observational cohort study of patients aged over 65 years without a diagnosis of dementia presenting for planned hip and knee replacements prior to the SARS-CoV-2 pandemic [approved by Office for Research Ethics Committee for Northern Ireland (REC ref: 10/NIR01/5)]. All methods were performed in accordance with the relevant guidelines and regulations and all participants signed an informed consent form. The study methodology has been described in detail previously^11,13^. Briefly, patients aged 65 years or older entering the elective hip or knee arthroplasty to a single surgical centre were eligible for inclusion. Exclusion criteria included a pre-existing diagnosis of dementia or other neurodegenerative condition. Participants were assessed for delirium daily postoperatively by a single researcher for the first three days. Confusion Assessment Method (CAM) was used for the delirium diagnosis. The incidence of postoperative delirium was 14%. Cohort had a mean age of 74.4 years and 57% were female. Paired CSF and blood metabolomics analysis was undertaken for 54 age and gender matched patients where 28 of the patients experienced post-operative delirium (based on CAM test) and 26 did not show any delirium symptoms (control). Metabolomic analysis was undertaken where sufficient CSF was available for age and gender matched delirium and control participants. General patient cohort characteristics are shown in Table 1 indicating Mini-Mental State Exam (MMSE) as one of the measures of preoperative dementia with score of 20 to 24 suggesting mild dementia, 13 to 20 moderate dementia, and less than 12 indicating severe dementia. Power analyses, performed in MetaboAnalyst for both CSF and Blood sample separation of control and delirium groups, showed normal distribution (following feature normalization) and presence of features with significant *p* values. Details of the sample selection of power analysis for this set were provided previously^11,13^.

**Table 1:** Patient characteristics in two cohorts showing matching in terms of age, comorbidities, as well as preoperative MMSE score.

Postoperative status	Age at surgery	Charlson comorbidity index	Surgery (hip/knee)	Number of regular medications	Preoperative MMSE score
Control	76 (66–87)	0 (0–2)	15/11	7 (0–12)	28 (23–30)
Post-operative delirium	76 (66–89)	1 (0–3)	9/19	6 (1–14)	26 (16–30)

### Metabolomics

The analysis of metabolic profiles of CSF samples from the nested case–control postoperative delirium cohort has previously been published^13^. In this investigation the corresponding blood plasma samples of each of the same patients were examined by an identical kit-based methodology. Quantitative metabolomic profiling was performed using the Biocrates AbsoluteIDQ p180 (BIOCRATES, Life Science AG, Innsbruck, Austria) using a Xevo TQ-MS triple-quadrupole mass spectrometer (Waters Corporation, Milford, USA) as previously described^26^. Briefly, this comprised of two general methods: UPLC (I-Class, Waters Corporation, UK) reversed-phase (Waters ACQUITY UPLC BEH C18 2.1 × 50 mm, 1.7 μm; UK) with multiple reaction monitoring (MRM), and flow injection analysis (FIA) also operating in MRM mode. Metabolite concentrations were calculated and expressed as micromolar (µM).

### SARS-CoV-2 metabolomics dataset

Plasma metabolomics profiles of SARS-CoV-2 patients were published and made available by Shen et al.^14^. The provided dataset includes metabolomic analysis of serum samples from patients with mild or severe COVID-19 as well as control subjects [with number of patient equal to severe (28), mild (37) and control (28)]. Measurements of CSF samples is not available for this cohort. In the original study COVID-19 patients are classified into mild or severe subgroups as: (a) mild symptoms without pneumonia; (b) severe: fulfill any of the three criteria: respiratory distress, respiratory rate R 30 times/min; means oxygen saturation % 93% in resting state; arterial blood oxygen partial pressure/oxygen concentration % 300 mmHg (1 mmHg = 0.133 kPa). Briefly, authors used the ultra-performance liquid chromatography/tandem mass spectrometry (UPLC-MS/MS) for untargeted metabolomics of serum samples providing identification and quantification of 941 metabolites including 36 drugs and their metabolites. Details of methodology and validation are provided in the original publication^14^.

### Data analysis and protein simulations

Different machine learning and statistical methods running under Matlab 2020 (Matworks Inc), Orange 3.25^27^, TMeV^28^ and Python—Jupyter Notebook were used for the analysis of metabolomics data. Principal component analysis (PCA) and 2-class Partial Least Squares-Discriminant Analysis (PLS-DA) model investigation was performed using jupyter notebook on data that was log10 transformed with selection to have an under 20% standard deviation and fewer than 10% of missing values in measurements for all samples. Selection of major features in different groups was performed using Significant Analysis of Microarrays (SAM)^29^. Machine learning analysis was performed using Python and Matlab with the results of Python Random Forest classification with SHAP (SHapley Additive exPlanations) algorithm shown to explain the output of machine learning^30^. SHAP routine was adapted from^31^.

Feature selection with Random Forest method was performed using a robust probabilistic method for our sample in order to augment our training set and, at the same time, utilize the actual errors associated with the measurements. Errors were estimated at 2 percent of the absolute value of measurements. Here, we adopt a Gaussian function with the standard deviation equals the errors (i.e., sigma = 0.02 × the measurement). Considering these errors, random sampling of the function should provide acceptable values. In this way, we have augmented our training set significantly. This data set is then randomly separated into a training set (75%) and a validation set (25%) to control overfitting. The uncertainties, including the size of the sample, are reflected in the error bars. The Random Forest procedure was as follows: (1) The most effective feature among all the features (metabolites) is found by RF; (2) The 'first' most effective feature is recorded and then eliminated from the table; (3) Remaining features are used to find the second most effective parameter among the subgroup. (4) This method is repeated to find the rest of the important features providing ranking for all features. RF was performed with bootstrap sampling. In order to obtain optimal probabilistic results, the method is repeated 100 times to obtain 100 different results providing distribution for each feature. Gaussian function is subsequently fitted to the distribution, and the final metabolite ranking is obtained from the mode of the fitted function. The error in the ranking, for a selected parameter, is estimated from the scatter (one sigma) of the function. This method is implemented within Jupyter Python notebook.

Correlation between metabolites in the CSF and blood was determined using Distance correlation using function *distcorr* in Matlab^32^. Distance correlation provides information about linear and non-linear correlation there-by giving insight into both direct and indirect feature correlations. Distance correlation analysis was utilized in order to explore differences in metabolite behaviour across the blood–brain-barrier in the two patient groups. Correlation was calculated between each metabolite in CSF and all metabolites in blood providing correlation measures across the blood brain barrier. Metabolites are selected based on the change in the distance correlation between the two subject groups determined by performing linear regression analysis of distance correlation for a metabolite in delirium-prone group vs control group. Significant change in correlation between control and delirium-prone group was obtained using linear regression analysis (using *regression* function in Matlab) between distance correlation values for each metabolite in CSF relative to all other metabolites in blood. Metabolites showing negative correlation (regression R < 0) are shown as significantly changed.

Protein sequences off MAOB, ACE2 and SARS-CoV-2 Spike protein were obtained from UniProt database and compared using Clustal Omega (https://www.ebi.ac.uk/Tools/msa/clustalo/). A comparison of the structures of MAOB and ACE2 bonded to SARS-CoV-2 Spike proteins are performed using UCSF Chimera, Schrodinger software package (Schrodinger Inc.) and PDB Data Bank structure analysis methodologies including jFATCAT^33^. Protein X-ray structures were obtained from https://www.rcsb.org/ and included for MAOB protein PDB ID 1GOS^34^ and ACE2 bonded to SARS-CoV-2 Spike protein PDB ID: 6M0J.

Computational analysis of the spike-MAOB docking was performed using Schrodinger’s BioLuminate package with spike protein obtained from 6M0J PDB entry was used as ligand and each MAOB chain obtained from 6FWC PDB entry was used as a receptor. All protein structures were optimized using Maestro (Schrodinger Inc.) using OPLS3e force field after addition of hydrogens^35^. The opposite analysis with MAOB chains as ligands and Spike protein as receptor led to the same optimal result. MAOB membrane bound regions have been excluded in docking calculations however effect of membrane was not considered. Docking calculation was performed 70,000 times and docking location with potential energy calculated using OPLS3e. The docking orientation with the lowest potential energy and without steric interference with the membrane is selected and shown.

## Results

### Detailed metabolomic analysis shows differences in delirium-prone and delirium-free patient groups prior to surgery in both blood and CSF metabolites

Relative concentrations of metabolites in control and delirium-prone patient cohorts obtained in CSF and blood samples are shown as a heat map in Supplementary Fig. 1. Initial data assessment was performed using Principal component analysis (PCA) and 2-class Partial Least Squares-Discriminant Analysis (PLS-DA) were performed on CSF and blood metabolomic data samples. For both sample types PCA achieved only limited separation of control and delirium-prone cases (Supplementary Fig. 2A,B). Incorporation of patient gender had very minor improvement of PCA group separation (Supplementary Fig. 2E,F). Supervised PLS-DA identified combination of metabolite features capable of clearly distinguishing the two patient groups (Supplementary Fig. 2C,D). For the CSF data the output was closely aligned with the previously published analysis^13^. The importance of specific metabolites to the model's discriminatory power are shown in coefficient plots (Supplementary Figs. 3–5). The most important contributors in control and delirium sample separation in CSF were spermidine, putrescine, glutamine (as previously reported by Pan et al.^13^), but also 3-hydroxypalmitoleylcarnitine (C16-1-OH), linoleic acid (C18-2), carnitine (C0) and, to a lesser extent, a number of other metabolites (Supplementary Fig. 3 shown in red). For blood metabolomic data the discrimination of control and delirium patients was influenced most by levels of proline, ornithine, lysine, trans-4-hydroxyproline (t4-OH-Pro), PC aa C24:0 and PC aa C26:0 as well as H1, ADMA, C7-DC and, to a lesser extent, a number of other metabolites (shown in red in Supplementary Fig. 3 with the most significant metabolites selected using PLS-DA with c.v. and SAM showing in Supplementary Figs. 4 and 5).

**Figure 1:**
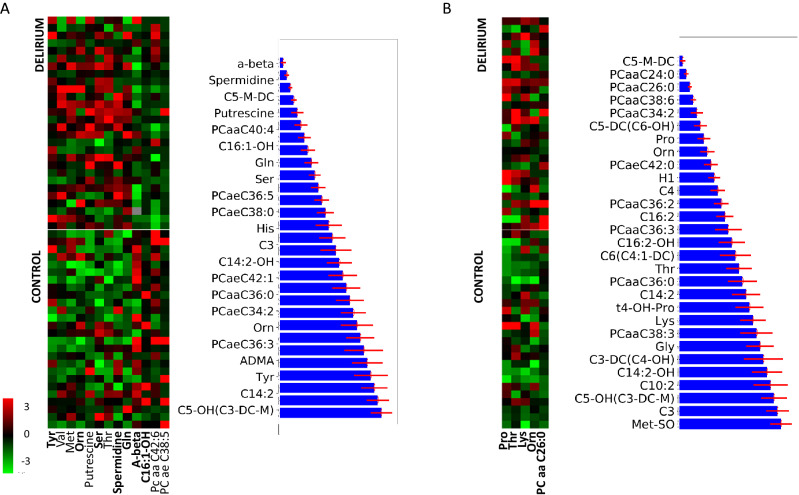
Most significant features distinguishing between control and delirium-prone cohort in (**A**) CSF and (**B**) Blood. SAM subset includes the most relevant metabolites based on statistical analysis (with delta value in TMeV implementation of SAM of 0.5) while the Random Forest method ranks metabolites’ relevance for diagnosis between two groups. Metabolic panel was selected from the Random Forest ranking Error bars shown in red represent uncertainties measure obtained using the probabilistic bootstrap method (see Materials and Methods for more details).

The above results were further corroborated by application of Statistical Analysis for Microarrays (SAM)^29^, which has been previously applied to metabolomics data^36^ (Fig. 1A). SAM analysis of normalized CSF data found significant differences in CSF for ornithine, glutamine, putrescine, spermidine and threonine, similarly as previously shown^13^ and in good agreement with the above PLS-DA (ornithine, glutamine, putrescine and spermidine were major features). Threonine was not one of the most significant contributors to the classification coefficients, but was still a significant contributor to the PLS-DA sample grouping. SAM analysis of blood metabolites shows major differences in pre-operative blood samples in proline, threonine, lysine, ornithine and phosphatidylcholines (specifically PC aa C26:0).

**Figure 2:**
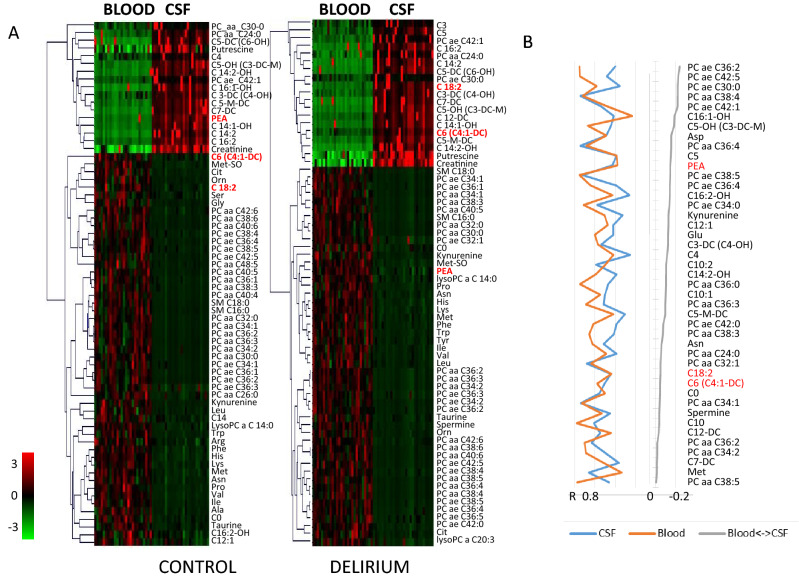
(**A**) SAM analysis of major differences between CSF and blood metabolites in the control and delirium groups. In both cases analysis was performed following metabolite and sample normalization in TMeV. For PEA—Control, adjusted p-value 1.8E-6; for PEA—Delirium, adjusted p-value 1.78 E-10. (**B**) Distance correlation between metabolites within CSF, within blood and between blood and CSF. Presented metabolite order shows the most significant, opposite regression between distance correlations of metabolites in blood to metabolites in CSF in control and delirium-prone cohorts.

Random forest (RF) is a supervised learning algorithm used here for the selection of significant metabolic features for classification between two patient groups. RF method builds an ensemble of decision trees that is in this application used to determine significance of each feature for the classification problem. Panel of the most diagnostically significant features is shown in Fig. 2. Selected panel of metabolites is shown together with the obtained classification rank and error in the rank based on the number of times the metabolite is listed with different rank in the repeated sampling process. Majority of SAM selected CSF metabolites and all metabolites selected in blood are also selected amongst the RF panel highest ranking group (shown in bold in Fig. 1).

### Phenethylamine, octadecadienylcarnitine and hexanoylcarnitine show opposite concentration difference in blood and CSF in delirium-prone and delirium-free patient groups

The availability of metabolic profiles for CSF and blood provides a unique possibility for the determination of significant differences in metabolite concentrations between these two body fluids. Comparison of concentrations in CSF and blood can provide information about the transfer and metabolism across the blood brain barrier as well as the possibility for the determination of blood-based biomarkers that are representative of changes in the CSF (Fig. 2). As can be expected there are major metabolic differences between CSF and blood in both control and delirium-prone groups with the majority of metabolites showing highly comparable behaviour in the two subject groups. Notable exceptions are metabolites that show opposite relative concentration difference in the two body fluids namely—phenethylamine (PEA) with higher concentration in CSF relative to blood in control subjects and opposite in delirium and Octadecadienylcarnitine (C 18:2) and hexanoylcarnitine [C6 (C4:1-DC)] showing higher concentration in blood than in CSF in control group. PEA is a natural monoamine alkaloid that acts as a central nervous system stimulant. Octadecadienylcarnitine (also called Linoleyl carnitine) and hexanoylcarnitine are long-chain acyl fatty acid derivatives of carnitine. Differences in concentrations in these three metabolites are significant in both control and delirium groups (following z-score normalization of metabolites and scaling of combined CSF and blood samples) with adjusted p-values for blood to CSF groups in control: for PEA adj. p = 1.83e−6; C 18:2 adj. p = 6.1e−4 and C6 (C4:1-DC) adj. p = 6.6e−4 and delirium: PEA adj. p = 1.8e−10; C 18:2 adj. p = 2.8e−10 and C6 (C4:1-DC) adj. p = 5.9e−8. However, the difference in their concentrations between control and delirium groups observed separately in CSF and blood is only minor. In CSF PEA is overall slightly reduced in the delirium group (adj. p-value = 0.1) while C 18:2 and C6 (C4:1-DC) are unchanged. Similarly in blood PEA concentration is slightly lower in delirium and C 18:2 and C6 (C4:1-DC) are unchanged.

Behaviour of metabolites in the two patient cohorts was further investigated using distance correlation analysis^31^. Major changes in the behaviour of metabolites is selected based on linear regression analysis of distance correlation for a metabolite in delirium-prone group vs control group. Selected in Fig. 2B are metabolites with negative regression coefficient suggesting major differences in the correlation network between two cohorts. Comparison of distance correlations for each metabolite for two patient cohorts are shown as scatter plot in Supplementary Fig. 6. Metabolites that, based on the regression analysis of distance correlations, show opposite behaviour in control and delirium-prone cohort include (Fig. 2B) several acylcarnitines, glycerophospholipids, amino acids and polyamines including PEA and (C 18:2) and C6 (C4:1-DC) that are observed to have opposite relative concentration across CSF and blood in the two patient groups. Proteins and other metabolites directly related to PEA and (C 18:2) and C6 (C4:1-DC) obtained from Metabolic Atlas^37^ are shown in Fig. 2.

**Figure 3:**
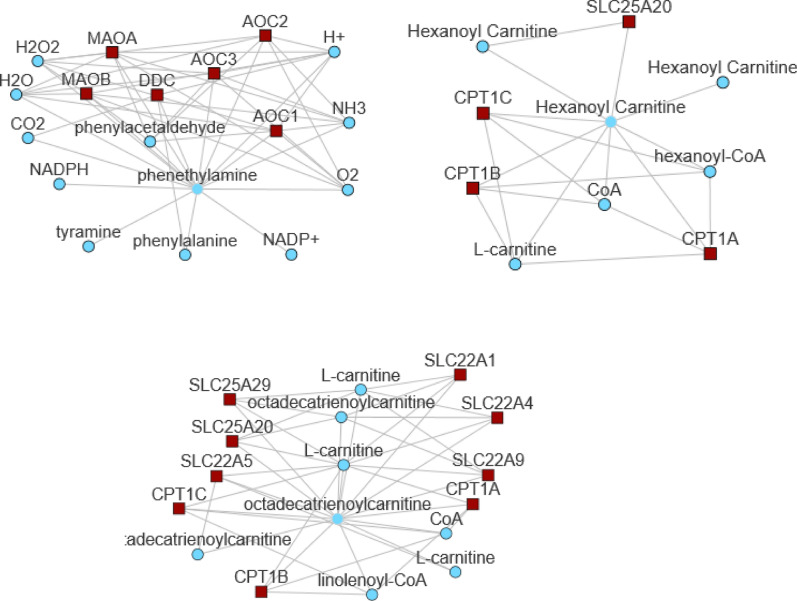
Metabolic Atlas analysis of interaction partners for PEA and carnitines. Many metabolites determined to be significantly altered in delirium-prone patients are closely linked to these three metabolites based on the metabolic network^37^. Figure is obtained from metabolicatlas.org^37^.

Many of the proteins presented in Fig. 3 have been previously associated with Alzheimer’s disease phenotype and include enzymes (e.g. monoamine oxidases—MAO A and B, Amiloride-sensitive amine oxidase AOC1, 2 and 3, Carnitine Palmitoyltransferase—CPT1A,B and C) as well as number of transporters including SLC22A1, 4, 5 and 9; SLC2520 and 29. MAO catalyzes the oxidative deamination of amines with a major function in the metabolism of neuroactive and vasoactive amines. AOC proteins are involved in the degradation of compounds such as putrescine, histamine, spermine, and spermidine. CPT proteins support transfer of the acyl group of long-chain fatty acid-CoA conjugates onto carnitine playing a major role in mitochondrial uptake of long-chain fatty acids and their beta-oxidation.

**Figure 4:**
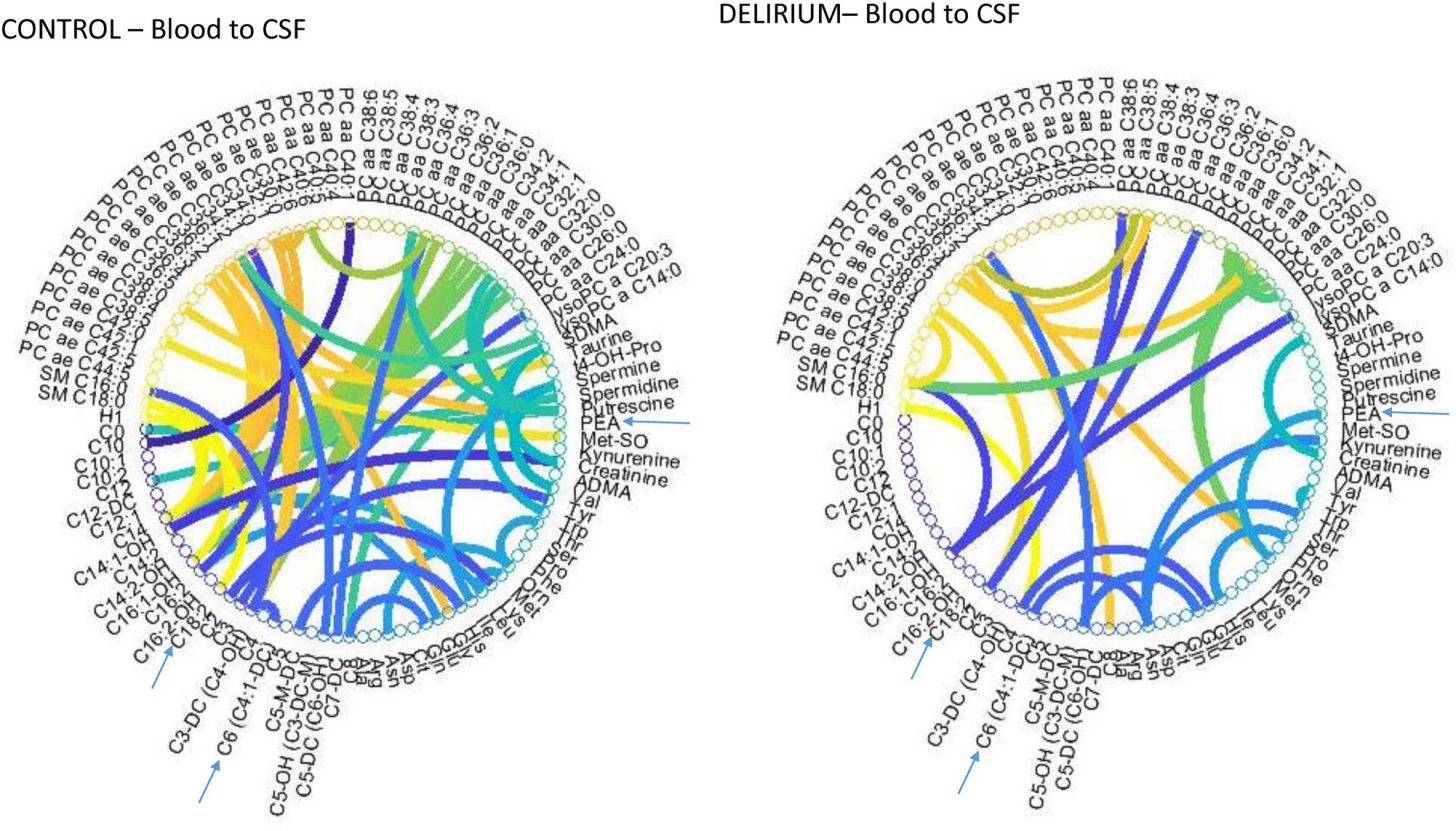
Distance correlation between metabolites in CSF and Blood in control and delirium-prone groups. Shown are correlations over 0.58. Figure is obtained using Matlab 2021a (Mathworks) Inc with cirularGraph (https://github.com/paul-kassebaum-mathworks/circularGraph).

Further analysis of the interaction between metabolite in CSF and blood is observed through the analysis of correlation network obtained using distance correlations. This metabolic network across the blood brain barrier is visualized in Fig. 4.

Number of metabolites show different interaction networks in the control and delirium-prone cohort. Differences in correlations are specifically indicated for three metabolites showing opposite relative concentration in CSF and blood with PEA for example showing strong correlation with ornithine only in the delirium-prone cohort. Carnitine derivatives as well as number of glycerophospholipids also show significantly different correlation partners across the blood–brain-barrier in the delirium-prone group indicating activation of different metabolic pathways in the two patient groups.

### MAO oxidizes PEA and changes in MAO function can explain observed differences in PEA in the two patient groups

Observed changes in PEA levels suggest changes in activity of MAO in delirium-prone patient cohort. MAOB is the only MAO protein found in platelets (for MAOA and MAOB protein expression please see Supplementary Fig. 7). Other enzymes linked to PEA metabolism (Fig. 2) show significantly lower level of expression in tissues with particularly low expression in nervous system and thus cannot account for the change in PEA concentration in CSF (Supplementary Fig. 8).

### Analysis of published metabolomics data for COVID-19 patients shows significance difference in several metabolites that can be related to the changes in MAO function

In order to determine relationship between MAO and the related metabolites to the severity of SARS-CoV-2 response we have explored the dataset provided by^14^ initially analysing the major metabolic differences between mild and severe COVID-19 cases. Patient information provided with the original publication does not include any data on possible delirium in these patients and we assumed, based on recent clinical studies^38^ that patients with severe disease were more likely to have delirium than mild cases (with 60–70% in severe cases and 20–30% of hospitalized patients). For comparison we are providing major features selected using statistical, SAM methods (Fig. 2A) as well as machine learning—Random Forest methodology and SHAP analysis of feature (metabolite) contribution to the ML model (Fig. 2B).

**Figure 5:**
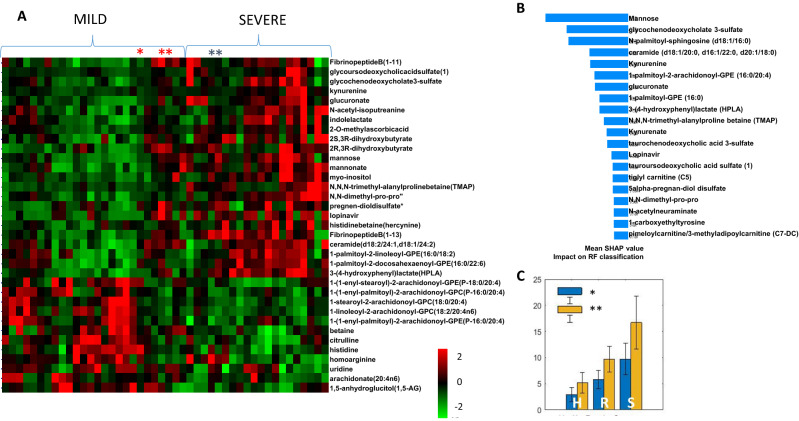
Analysis of major metabolic differences between Mild and Severe COVID-19 cases based on^14^ dataset. (**A**) SAM analysis of major features following sample and metabolite normalization; (**B**) SHAP analysis of major contributors to Random Forest classification of mild *vs* severe cases. (**C**) Median value and standard deviation of ratios between metabolites known to either activate MAO for healthy (H), mild (R) and severe (S) COVID-19 cases. Shown is the ratios of *ceramide (d18:1/20:0, d16:1/22:0, d20:1/18:0)/sphingosine 1-phosphate and **ceramide (d18:2/24:1, d18:1/24:2)/sphingosine 1-phosphate.

The number of metabolites showing major concentration difference between severe and mild cases can be related to pathways involving MAO enzyme (see Supplementary Fig. 9 for all known direct interactions of MAOA and/or MAOB and metabolites). Specifically ratios of ceramide to sphingosine 1-phosphate (known as “sphingolipid rheostat”) is known to affect activity of MAO^39^. The ratio between these metabolites based on the COVID-19 patient data provided by^14^ is shown in Fig. 5C and shows an increase with disease severity suggesting increasing activity of MAO.

### Major structural similarity between region of MAOB and ACE2 region binding to SARS-CoV-2 spike protein suggests potential for MAOB-spike protein interaction

In order to investigate the possibility that the SARS-CoV-2 virus directly influences MAOB function the structures of MAOB and ACE2 (as a known binding target of SARS-CoV-2) were compared^4^. Overall structural comparison between MAOB (PDB structure 1GOS) and ACE2 (PDB structure 6M0J), showed only 51% overall structural similarity. However the comparison of the ACE2—spike protein binding region with MAOB resulted in 90% to 100% structure overlap. Further computational analysis of the spike-protein relation to MAOB protein is shown in Fig. 5.

**Figure 6:**
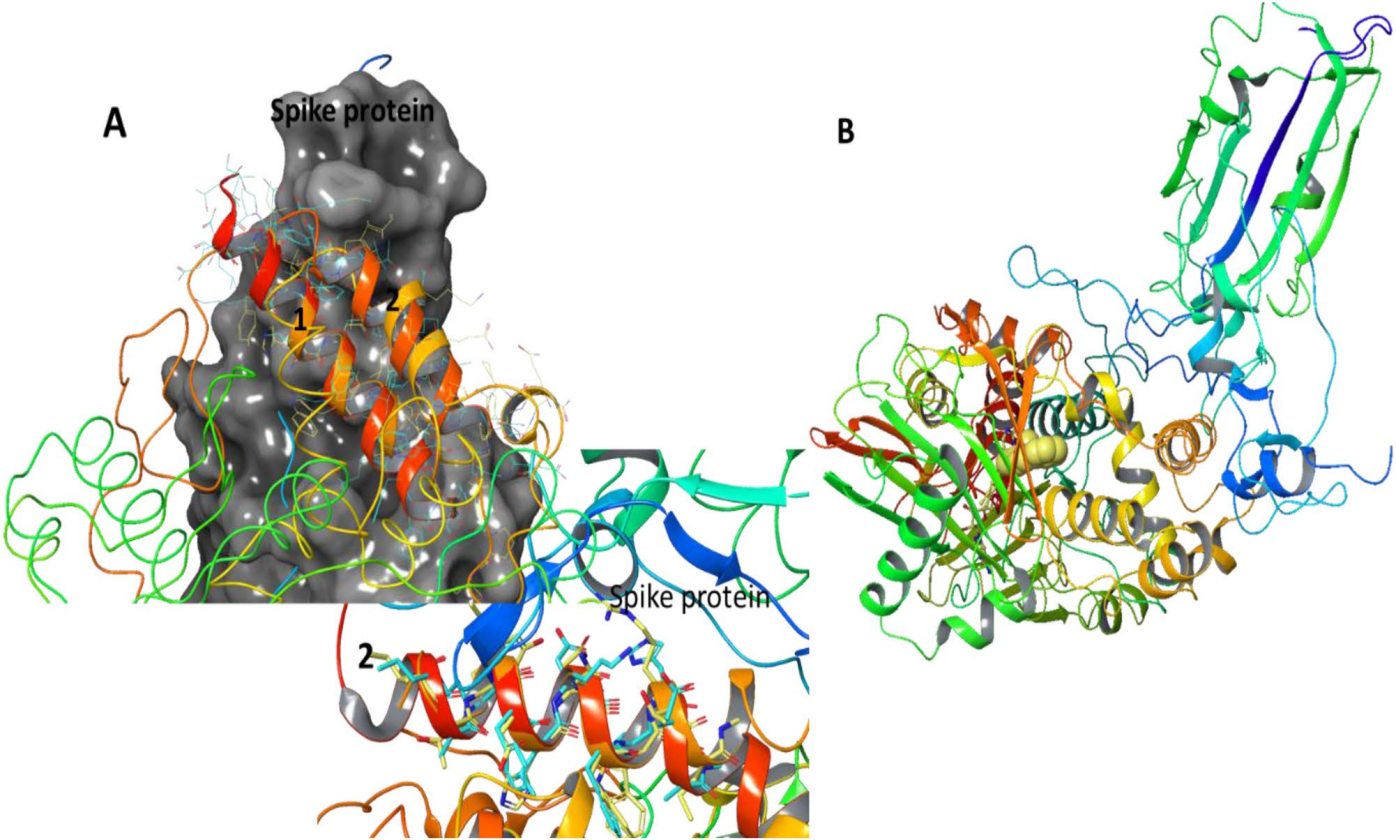
(**A**) Analysis of the overall structure similarity between ACE2 and MAOB using jFATCAT_flexible^33^ resulting in 6M0J Similarity: 42% 1GOS Similarity: 51% P-value: 6.67e−01. Further analysis of structure subsection structural alignment in the regions of ACE2 related to binding Spike protein are shown in 1 and 2 showing major similarity (section 1 6M0J Similarity: 100% 1GOS Similarity: 100% P-value: 1.67e−02 section 2 6M0J Similarity: 74%; 1GOS Similarity: 100% P-value: 5.01e−01); (**B**) Hypothetical binding between Spike protein and MAOB outlining the ligand location in the MAOB protein. Figure and analysis is done using Schrodinger 2020–2 (Schrodinger Inc.)^35^.

Specifically, analysis of the overall structure similarity between ACE2 and MAOB using jFATCAT_flexible^33^ resulted in ACE2 similarity of 42% and MAOB similarity of 51% with the P-value: 6.67e−01. However, further analysis of subsection of ACE2 involved in binding to Spike protein indicated as 1 and 2 (Fig. 6A) shows major similarity (section 1 6M0J Similarity: 100% 1GOS Similarity: 100% P-value: 1.67e−02 section 2 6M0J Similarity: 74%; 1GOS Similarity: 100% P-value: 5.01e−01) suggesting possibility for interaction between MAOB and SARS-CoV-2 spike protein (Fig. 6A shows the association between MAOB and ACE2 overlapping regions and spike protein) leading to possibility for MAOB activity change in severe COVID-19 patients (Fig. 6B shows computational representation of the binding location of spike protein to MAOB indicating also the MAOB ligand).

**Figure 7:**
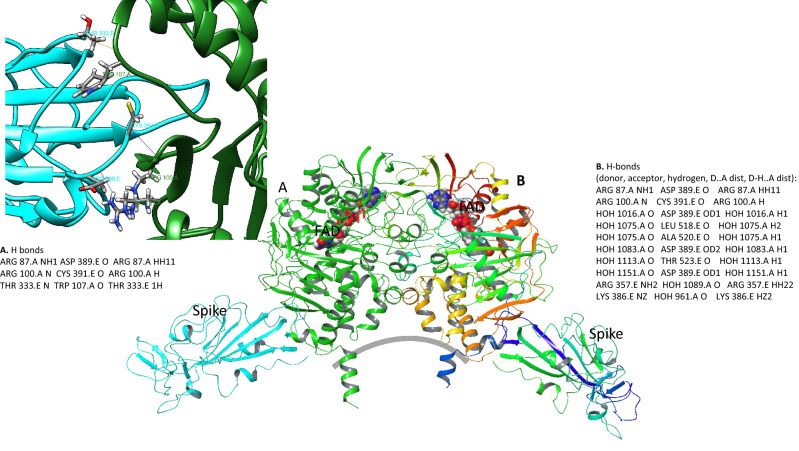
Computational Protein–Protein docking analysis of the energetically preferred binding location for Spike proteins to MAOB (Schrodinger Inc.). Approximate location of the bilayer is indicated with a gray line. Binding energy calculation was performed for 70,000 consecutively selected positions of Spike protein as ligand and each chain of MAOB as a receptor. Shown are Spike proteins, MAOB chains as well as FAD—flavin adenine dinucleotide cofactor. Figure and analysis is done using Schrodinger 2020–2 (Schrodinger Inc.)^35^.

Protein–protein docking analysis provides theoretical information about the most energetically beneficial binding orientation between proteins. In this analysis we have explored preferred arrangement with each chain of MAOB dimer acting as a receptor and SARS-CoV-2 spike protein viewed by the software as a ligand and *v.v*. Rigorous selection of the most energetically stable docking pose was selected amongst 70,000 different orientation of receptor and ligand proteins and calculated binding energy. The most stable docking form, determined from the lowest potential energy of the system is shown in Fig. 7. The MAOB membrane bound residues have been excluded in the calculation of energy. Membrane is added subsequently in order to select structures with no steric constraints. Final selection of the optimal docking arrangement is shown in Fig. 7 with information about predicted hydrogen bond partners for solvent free (A) and with water cloud (B).

Potential energy of binding between spike proteins and two MAOB chains is calculated using OPLS3 force field. For Chain A docking analysis was performed without solvent and the lowest energy of binding obtained in the docking analysis was for structure with energy of − 13,424.67 kcal/mol, For chain B we included a solvent cloud in calculating docking energies. The lowest configuration in the region outside of the membrane had potential energy of − 19,073.44 kcal/mol and is shown in Fig. 7. Hydrogen bonding partners are listed for both dimers and show that docking structure B has the same binding partners between Spike protein and MAOB in addition to hydrogen bonding to solvent. Resulting structure shows possible interference of spike protein with the recently presented membrane mediated substrate entrances to MAOB active site^40^.

## Discussion

Delirium is an acute change in attention, awareness and cognition occurring as a result of precipitants including medications, substance abuse, illness or surgery. Even routine surgical procedures, such as arthroplasty, are known to lead to post-operative delirium in a subset of patients with estimates of approx. 17% of elderly patients experiencing delirium after a routine surgery^12^. A growing body of evidence links SARS-CoV-2 infection to delirium incidence^15^. This investigation generated and interrogated metabolomics data in order to identify the underlying metabolic perturbations associated with post-operative delirium and then explored possible SARS-CoV-2 related links to these pathways.

A range of metabolite associations emerged through the application of a variety of data mining methods. In CSF both statistical analysis and the RF ranking of top diagnostic biomarkers (Fig. 1A) indicate major differences in the concentrations of: spermidine, putrescine, ornithine, tyrosine, glutamine, serotonin and hydroxyhexadecenoylcarnitine (C16:1-OH in figures). RF analysis also presented diagnostic relevance for several other metabolites with panels of 22 in CSF including spermidine, putrescine, ornithine and glutamine directly linked with arginine metabolism, all of which were higher in delirium-prone CSF samples.

The important features identified in blood and CSF can be grouped as either polyamines, acetylcarnitines or glycerophospholipids. RF ranking determined 3-methylglutarylcarnitine (C5-M-DC) as one of the most significant features in both blood and CSF. Serum levels of acetylcarnitines have previously been shown to progressively decrease from healthy through subjective memory complaint, mild cognitive impairment, up to Alzheimer’s disease (AD)^41^. Lower concentrations of several acetylcarnitines observed here in the delirium-prone group is potentially supportive of the view that delirium raises AD risk. Acetylcarnitine is associated with altered monoamine neurotransmitter levels suggesting possible function as a monoamine oxidase (MAO) inhibitor^42^. Interestingly, MAO is involved in the metabolism of polyamines, some of which were significantly altered in the delirium-prone group and in particular PEA.

PEA was one of the most profoundly affected metabolites in patients who later experienced postoperative delirium. In delirium-prone individuals PEA concentrations are slightly reduced both in the CSF and blood plasma relative to the control group. Interestingly, for PEA an inverse relationship exists in these two compartments for these two groups with the significantly smaller PEA concentration in CSF relative to blood in delirium-prone cohort. Brain monoamines include common biogenic amines (dopamine, norepinephrine, and serotonin) and trace amines such as PEA^43^. PEA has been shown to alter the serotonin transporter by interacting with trace amine-associated receptor 1 (TAAR1)^43^. Activation of TAAR1 with PEA significantly inhibits uptake and induces efflux of dopamine and norepinephrine.

Observed alterations in PEA are highly plausible because monoamine oxidase (MAO), one of the key enzymes responsible for its metabolism, is a known target for treatment of a variety of neurological conditions including depression, Parkinson’s disease and recently AD^18^. MAO is an enzyme localised on the outer mitochondrial membrane and it preferentially degrades benzylamine and PEA. The MAO family of proteins oxidizes a number of different amine substrates including small-molecule monoamines, polyamines as well as modified amino acids in proteins, and directly influences number of different metabolites (Supplementary Fig. 9). At the same time MAO proteins have recently been listed as the most affected by inhibitory action of metabolites with 1017 metabolites possibly inhibiting MAO function^44^.

Two MAO subtypes exist: monoamine oxidase A (MAOA) which preferentially oxidizes biogenic amines such as 5-hydroxytryptamine (5-HT), norepinephrine and epinephrine, and monoamine oxidase B (MAOB) which performs oxidative deamination of biogenic and xenobiotic amines. MAOB is particularly important for the metabolism of neuroactive and vasoactive amines in the central nervous system and peripheral tissues. Expression of MAOB increases with age and is associated with increased free radical damage and ROS formation. This in turn leads to a decrease in neuronal mitochondrial function and ultimately neurodegeneration^44^ which is partly due to reduced PEA concentrations.

MAO inhibitors have been extensively developed and utilized for treatment of depression^45^ (https://www.nice.org.uk/). A number of publications have also investigated the therapeutic effects of MAO inhibitors for other neurological conditions, such as AD, Parkinson’s disease, and depression^18^. Specifically, MAOB has been proposed as a possible therapeutic target for AD due to its association with aberrant GABA production, but it also has therapeutic relevance for Parkinson’s disease due to its role in dopamine depletion^18,46^.

Utilization of MAO inhibitors (MAOI) for depression treatment has resulted in a number of side effects including: agitation, irritability, ataxia, movement disorders, insomnia, drowsiness, vivid dreams, cognitive impairment, and slowed speech, hallucinations and paranoid delusions (https://www.nice.org.uk/). Additionally, MAOI has been linked to an increasing suicide, pyrexia, delirium, hypoxia, hypertension and fatal intravascular coagulation^47–49^. MAOB is highly expressed in neurons, as well as platelets^50^ possibly explaining the observed effects of MAOB inhibitors on neurological state as well as blood based complications (Supplementary Fig. 7 shows protein expression of MAOA and MAOB in different human tissues). MAOB has been linked to the activity of platelets and dysfunction of nitric oxide synthase pathway observed in number of neurological diseases (recently reviewed in Leiter and Walker^17^).

Differences in the activity of MAOB in the surgical delirium-prone patient population was indicated by a change in the concentration of PEA as well as polyamines. Observed changes in the concentrations of carnitine derivatives in this patient cohort can be linked with previously established relationship between acetyl l-carnitine and dopamine through its effect on MAOB expression in the presence of anesthetic^37^.

The over-activity of MAOB can result in the observed PEA concentration decrease and the major changes observed in the polyamines as well as related amino acids in the delirium-prone patients. Pro-inflammatory stimuli, including cytokines lead to MAO-dependent increases in reactive oxygen species causing mitochondrial dysfunction^16^.

Distance correlation analysis was utilized to explore differences in metabolite behaviour across the blood–brain-barrier in the two patient groups. Briefly, distance correlation assesses co-varying of features in two groups relative to their distances from all other points and provides a better method for the assessment of non-linear dependencies between variables. In our application distance correlation is expected to provide better method for assessing indirect relationships between metabolites including possible activities as inhibitors or activators of metabolic processes or transport.

Correlation analysis presented in Fig. 3B shows metabolites with the major change in their relationship between CSF and blood in the delirium-prone and control groups. Once again PEA shows a significantly altered correlation across the blood–brain-barrier with negative correlation between control and delirium-prone group as well as some specific differences in its interaction network. Metabolites showing opposite correlation between CSF and blood in the two groups show statistically more significant involvement with several pathways previously indicated as relevant in the AD development including: alanine, aspartate and glutamate metabolism (P = 7.916E−4), arginine biosynthesis (P = 0.0039794), Histidine metabolism (P = 0.0052069), beta-alanine metabolism (P = 0.0089), Glutathione metabolism (P = 0.015655), glycerophospholipid metabolism (P = 0.025), arginine and proline metabolism (P = 0.028), linoleic acid metabolism (P = 0.035028), nitrogen metabolism (P = 0.042), d-glutamine and d-glutamate metabolism (P = 0.042), phenylalanine metabolism (P = 0.068938). Major changes in the network between metabolite in CSF and in blood observed between control and delirium-prone cohort (Fig. 5) suggest possible changes in both metabolism as well as transport across the blood–brain-barrier with several possible protein targets indicated in Fig. 3 through their relation with the significantly changed metabolites in the two groups.

Previously reported links between HIV infection and changes in monoamine and acylcarnitine metabolites (as well as inflammatory markers) indicate that viral infection and inflammation can alter monoamine metabolism and mitochondrial energetics^51^. At the same time a number of side-effects previously listed for MAO inhibitors have also been observed in COVID-19 patients. One of the, as yet unresolved, effects of SARS-CoV-2 infection in a subgroup of patients is the development of a systemic coagulopathy and acquired thrombophilia characterized by a proclivity for venous, arterial and microvascular thrombosis^14^. Additionally, severe cases of SARS-CoV-2 infection (where delirium is common) also experience low blood oxygen levels, elevated urea, acute renal dysfunction^52^. Differences in the activity of MAOB in surgical delirium-prone patient population were indicated by a change in the concentration of PEA as well as polyamines. Observed changes in the concentrations of carnitine derivatives in this patient cohort can be linked with previously established relationship between acetyl l-carnitine and dopamine through its effect on MAOB expression in the presence of anesthetic^37^. Significant concentration increases in severely affected COVID-19 patients is observed for both indolacetate and kynurenine as well as apparent increase in metabolising of serotonin in severe cases (Fig. 4) all part of tryptophan metabolism utilizing MAOA. Additionally, tryptophan plasma concentration in both mild and severe patients is significantly reduced relative to the healthy subjects (Supplementary Fig. 10) corroborated with the reduction in the concentration of arachidonate and related metabolites in severe relative to mild and both groups of infected patients relative to healthy subjects. Arachidonic acid is metabolized by activated platelets possibly leading to their aggregation. High risk groups for severe response to SARS-CoV-2 infection have known increased activity of MAO enzymes including: age, obesity, diabetes, heart condition (www.who.int). In addition to other symptoms, SARS-CoV-2 causes hematological changes which include reduced platelet count^53^, platelet hyperactivity, changed gene expression in platelets (particularly in relation to protein ubiquitination), altered antigen presentation, and mitochondrial dysfunction in platelets^54^. Further analysis of the relationship between SARS-CoV-2 and platelets by Manne et al. have shown presence of mRNA of the SARS-CoV-2 N1 gene in isolated platelets from 2 ICU COVID-19 patients even in absence of ACE2 gene in these cells^54^. Additionally, SARS-CoV-2 mRNA was determined in platelets of COVID-19 patients in the recent study of Zaid et al.^55^ as well. Study by Zhang et al.^56^ shows that platelets expressed ACE2, a host cell receptor for SARS-CoV-2, as well as TMPRSS2, a serine protease for Spike protein priming and that the virus associates closely with platelets according to Transmission electron microscopy analysis in an ACE2 dependent manner. This study has also shown that SARS-CoV-2 and its Spike protein directly activates platelets and potentiates their prothrombotic function and inflammatory response. Furthermore, Real et al.^57^ shows that platelets from HIV infected patients can contain replication-competent HIV virus.

Given the known role of MAO in a number of SARS-CoV-2 symptoms this prompted us to perform a computational analysis examining SARS-CoV-2 virus spike protein interactions with MAO. The preliminary computational structure comparison of MAOB and ACE2 protein performed here determined whether there is a possibility that the SARS-CoV-2 spike protein binds to MAOB (Fig. 5). Although the sequence similarity between ACE2 and MAOB proteins is limited (see Supplementary Table for Clustal Omega sequence comparison) there is an almost coincident alignment and structural similarity in the region involved in SARS-CoV-2 spike protein binding (Fig. 7, Supplementary Fig. 11). It is also important to point out that similar comparison between structures of ACE2 and AOC1, AOC2, AOC3 showed less than 30% overall similarity and no significant structural overlap with the ACE2 binding region for spike protein. Binding of the spike protein to MAOB could result in a change in either its enzymatic function, its post-translational modification or association with is protein partners including cell surface amino oxidases such as vascular adhesion protein 1 (VAP-1) (also known as AOC3) a known non-classical inflammation-inducible endothelial molecule^58^.

The over-activity of MAOB can result in the observed PEA concentration decrease and the major changes observed in the polyamines as well as related amino acids in the delirium-prone patients. Pro-inflammatory stimuli, including cytokines lead to MAO-dependent increases in reactive oxygen species causing mitochondrial dysfunction^16^. At the same time, interference with MAOB activity in subjects with overactive MAOB can lead to the side-effects observed in MAOB inhibition as well as SARS-CoV-2 infection. The metabolomic data and symptom similarities of delirium prone patients from a surgical setting and COVID-19 patients indicate potential dysfunction in MAOB. Dexamethasone recently shown to improve survival in severe COVID-19 cases^59^ has been documented in the past to increase oxidative stress and the expression of MAOA^60^ and MAOB^61^ in dopaminergic neurons^62^. Computational modeling defines a mechanism by which the spike protein could directly bind to MAOB thereby interfering with its normal function and particularly affecting patients with increased MAOB expression. Detailed computational docking analysis shows strongest binding of spike protein to the region of MAOB recently proposed as the membrane mediated substrate entrance to the active site of MAOB^62^. Further analysis is currently under way to explore in greater detail the role of MAOB in delirium and SARS-CoV-2 infection with further exploration of the effects of sex and other demographic, medical or drug utilization on the delirium related metabolic changes.

## Conclusion

Significant differences were observed in a number of mono- and polyamines which led us to investigate in some detail the relationship between the observed changes in operative delirium and delirium caused by SARS-CoV-2 and to propose a hypothetical relationship between monoamine oxidase and the SARS-CoV-2 spike protein. Experimental analysis of the relationship between delirium and SARS-CoV-2 and the possibility for spike-protein binding to monoamine oxidase is currently underway. Further research is required to establish what effect MAOB inhibitors might have on these pathways. There is no evidence at present to support the withholding or increasing MAOB inhibitors in COVID-19 treatment.

## Supplementary Information

Supplementary Information.
