# Supplementary material for: Metabolomics and computational analysis of the role of monoamine oxidase activity in delirium and SARS-COV-2 infection
Source: Sci Rep. 2021 May 20;11:10629. doi: 10.1038/s41598-021-90243-1 (PMC8138024; doi:10.1038/s41598-021-90243-1)
Supplement: Supplementary file 1 — Supplementary Information. [file 41598_2021_90243_MOESM1_ESM.pptx]

## Slide 1
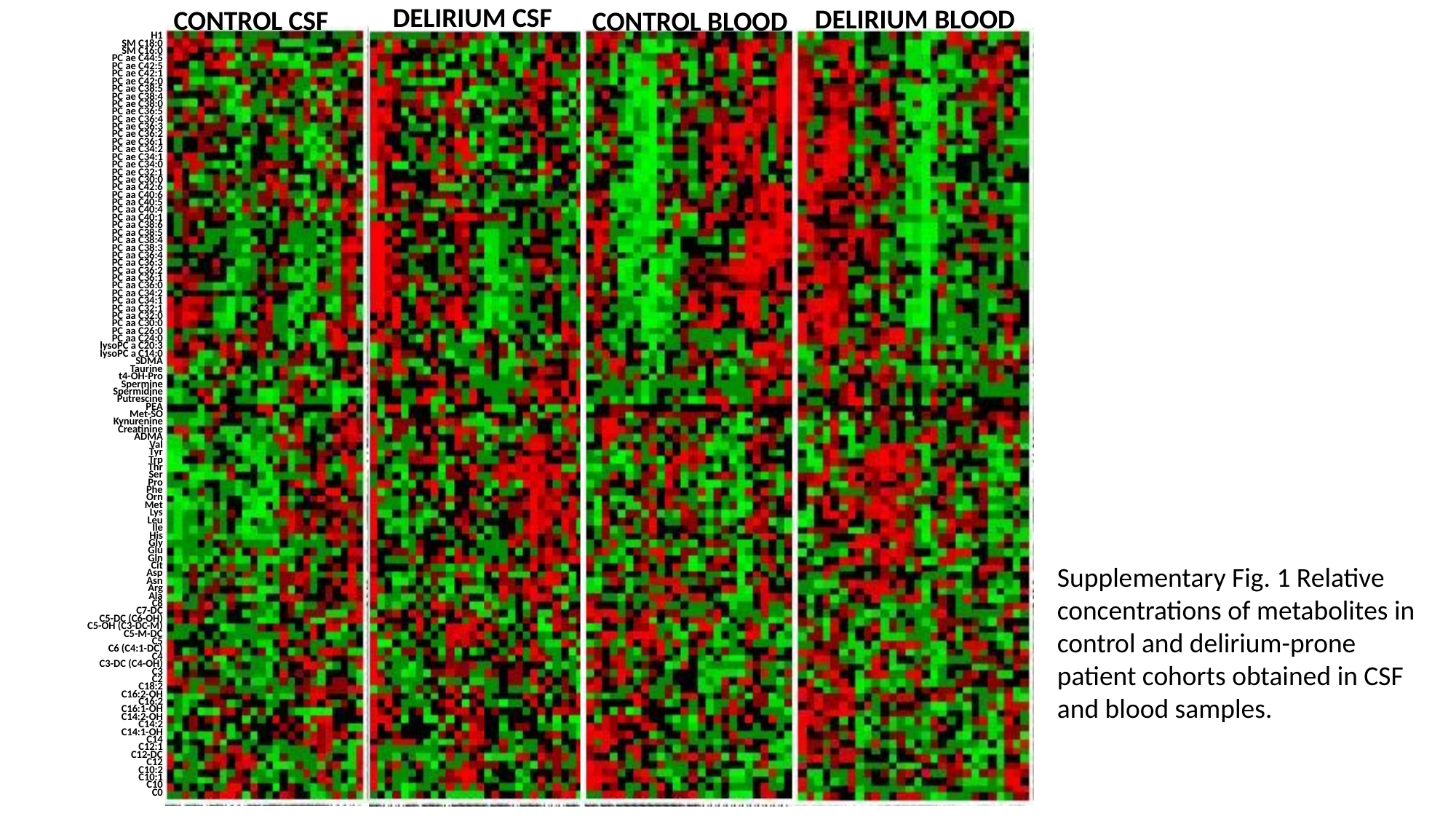

DELIRIUM CSF
DELIRIUM BLOOD
CONTROL CSF
CONTROL BLOOD
H1
SM C18:0
SM C16:0
PC ae C44:5
PC ae C42:5
PC ae C42:1
PC ae C42:0
PC ae C38:5
PC ae C38:4
PC ae C38:0
PC ae C36:5
PC ae C36:4
PC ae C36:3
PC ae C36:2
PC ae C36:1
PC ae C34:2
PC ae C34:1
PC ae C34:0
PC ae C32:1
PC ae C30:0
PC aa C42:6
PC aa C40:6
PC aa C40:5
PC aa C40:4
PC aa C40:1
PC aa C38:6
PC aa C38:5
PC aa C38:4
PC aa C38:3
PC aa C36:4
PC aa C36:3
PC aa C36:2
PC aa C36:1
PC aa C36:0
PC aa C34:2
PC aa C34:1
PC aa C32:1
PC aa C32:0
PC aa C30:0
PC aa C26:0
PC aa C24:0
lysoPC a C20:3
lysoPC a C14:0
SDMA
Taurine
t4-OH-Pro
Spermine
Spermidine
Putrescine
PEA
Met-SO
Kynurenine
Creatinine
ADMA
Val
Tyr
Trp
Thr
Ser
Pro
Phe
Orn
Met
Lys
Leu
Ile
His
Gly
Glu
Gln
Cit
Asp
Asn
Arg
Ala
C8
C7-DC
C5-DC (C6-OH)
C5-OH (C3-DC-M)
C5-M-DC
C5
C6 (C4:1-DC)
C4
C3-DC (C4-OH)
C3
C2
C18:2
C16:2-OH
C16:2
C16:1-OH
C14:2-OH
C14:2
C14:1-OH
C14
C12:1
C12-DC
C12
C10:2
C10:1
C10
C0
Supplementary Fig. 1 Relative concentrations of metabolites in control and delirium-prone patient cohorts obtained in CSF and blood samples.

## Slide 2
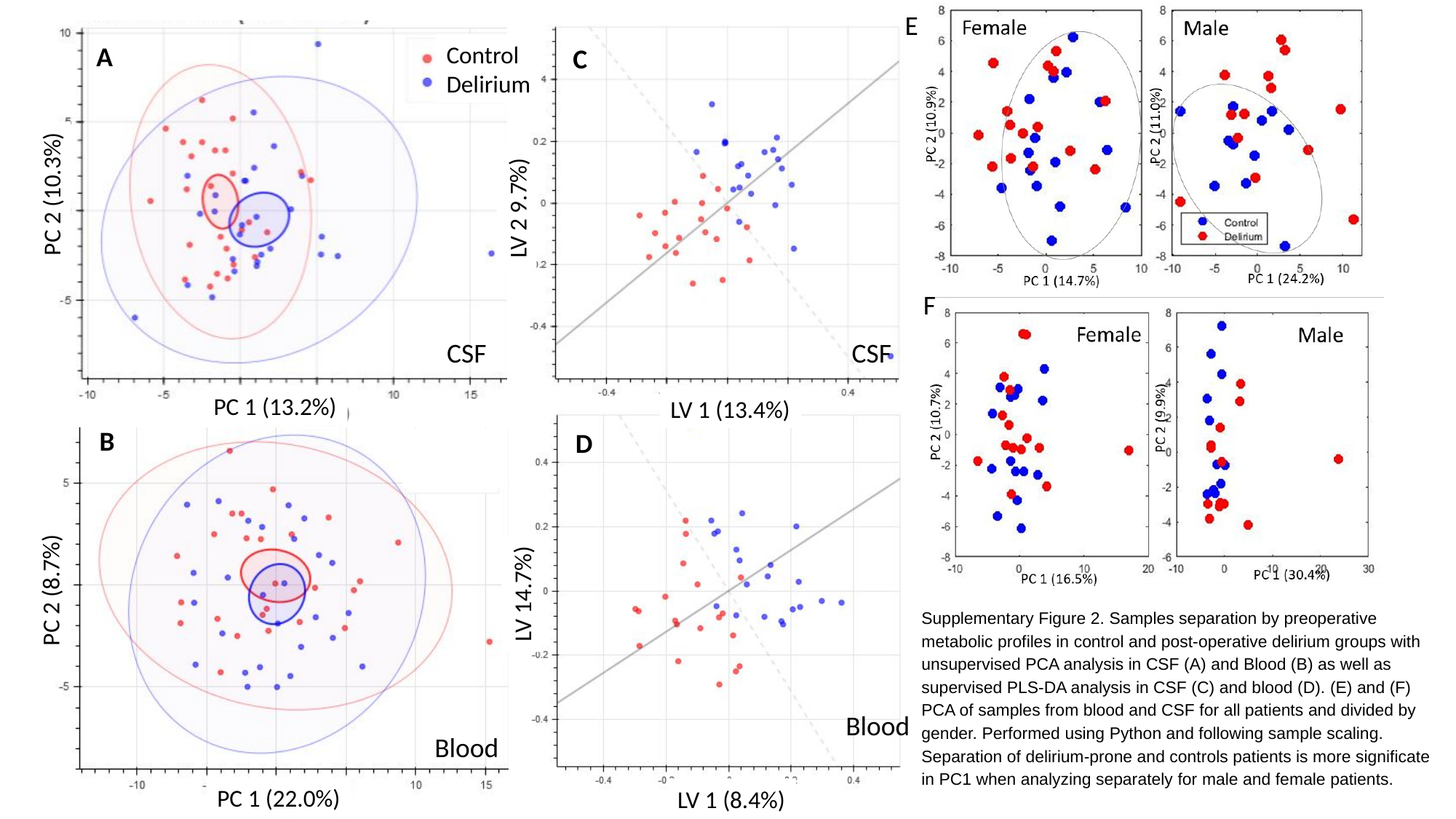

E
A
Control
Delirium
C
PC 2 (10.3%)
LV 2 9.7%)
CSF
CSF
PC 1 (13.2%)
LV 1 (13.4%)
B
D
PC 2 (8.7%)
LV 14.7%)
Blood
Blood
PC 1 (22.0%)
LV 1 (8.4%)
F
Supplementary Figure 2. Samples separation by preoperative metabolic profiles in control and post-operative delirium groups with unsupervised PCA analysis in CSF (A) and Blood (B) as well as supervised PLS-DA analysis in CSF (C) and blood (D). (E) and (F) PCA of samples from blood and CSF for all patients and divided by gender. Performed using Python and following sample scaling. Separation of delirium-prone and controls patients is more significate in PC1 when analyzing separately for male and female patients.

## Slide 3
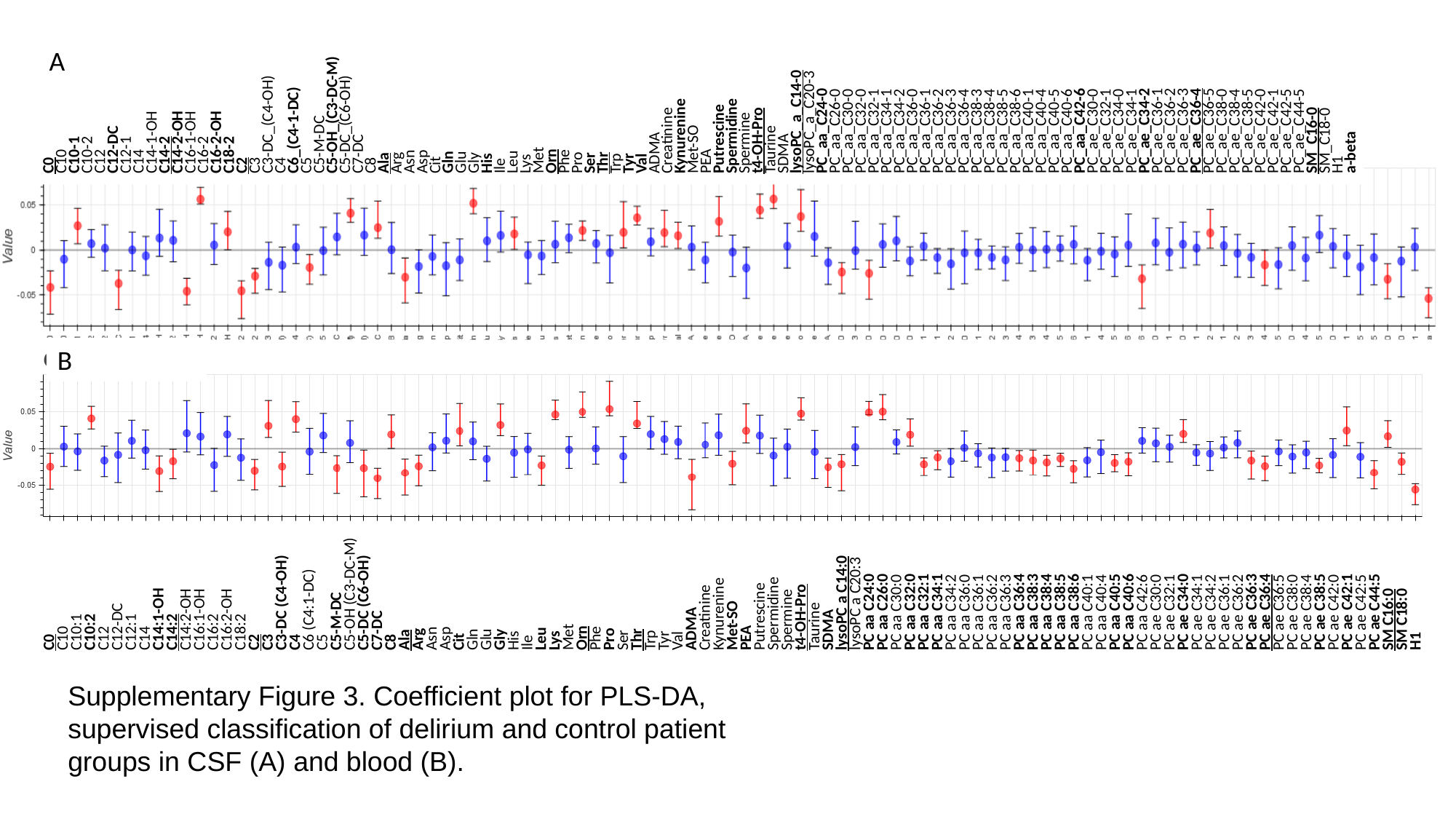

C0
C10
C10-1
C10-2
C12
C12-DC
C12-1
C14
C14-1-OH
C14-2
C14-2-OH
C16-1-OH
C16-2
C16-2-OH
C18-2
C2
C3
C3-DC_(C4-OH)
C4
C6_(C4-1-DC)
C5
C5-M-DC
C5-OH_(C3-DC-M)
C5-DC_(C6-OH)
C7-DC
C8
Ala
Arg
Asn
Asp
Cit
Gln
Glu
Gly
His
Ile
Leu
Lys
Met
Orn
Phe
Pro
Ser
Thr
Trp
Tyr
Val
ADMA
Creatinine
Kynurenine
Met-SO
PEA
Putrescine
Spermidine
Spermine
t4-OH-Pro
Taurine
SDMA
lysoPC_a_C14-0
lysoPC_a_C20-3
PC_aa_C24-0
PC_aa_C26-0
PC_aa_C30-0
PC_aa_C32-0
PC_aa_C32-1
PC_aa_C34-1
PC_aa_C34-2
PC_aa_C36-0
PC_aa_C36-1
PC_aa_C36-2
PC_aa_C36-3
PC_aa_C36-4
PC_aa_C38-3
PC_aa_C38-4
PC_aa_C38-5
PC_aa_C38-6
PC_aa_C40-1
PC_aa_C40-4
PC_aa_C40-5
PC_aa_C40-6
PC_aa_C42-6
PC_ae_C30-0
PC_ae_C32-1
PC_ae_C34-0
PC_ae_C34-1
PC_ae_C34-2
PC_ae_C36-1
PC_ae_C36-2
PC_ae_C36-3
PC_ae_C36-4
PC_ae_C36-5
PC_ae_C38-0
PC_ae_C38-4
PC_ae_C38-5
PC_ae_C42-0
PC_ae_C42-1
PC_ae_C42-5
PC_ae_C44-5
SM_C16-0
SM_C18-0
H1
a-beta
C0
C10
C10:1
C10:2
C12
C12-DC
C12:1
C14
C14:1-OH
C14:2
C14:2-OH
C16:1-OH
C16:2
C16:2-OH
C18:2
C2
C3
C3-DC (C4-OH)
C4
C6 (C4:1-DC)
C5
C5-M-DC
C5-OH (C3-DC-M)
C5-DC (C6-OH)
C7-DC
C8
Ala
Arg
Asn
Asp
Cit
Gln
Glu
Gly
His
Ile
Leu
Lys
Met
Orn
Phe
Pro
Ser
Thr
Trp
Tyr
Val
ADMA
Creatinine
Kynurenine
Met-SO
PEA
Putrescine
Spermidine
Spermine
t4-OH-Pro
Taurine
SDMA
lysoPC a C14:0
lysoPC a C20:3
PC aa C24:0
PC aa C26:0
PC aa C30:0
PC aa C32:0
PC aa C32:1
PC aa C34:1
PC aa C34:2
PC aa C36:0
PC aa C36:1
PC aa C36:2
PC aa C36:3
PC aa C36:4
PC aa C38:3
PC aa C38:4
PC aa C38:5
PC aa C38:6
PC aa C40:1
PC aa C40:4
PC aa C40:5
PC aa C40:6
PC aa C42:6
PC ae C30:0
PC ae C32:1
PC ae C34:0
PC ae C34:1
PC ae C34:2
PC ae C36:1
PC ae C36:2
PC ae C36:3
PC ae C36:4
PC ae C36:5
PC ae C38:0
PC ae C38:4
PC ae C38:5
PC ae C42:0
PC ae C42:1
PC ae C42:5
PC ae C44:5
SM C16:0
SM C18:0
H1
A
B
Supplementary Figure 3. Coefficient plot for PLS-DA, supervised classification of delirium and control patient groups in CSF (A) and blood (B).

## Slide 4
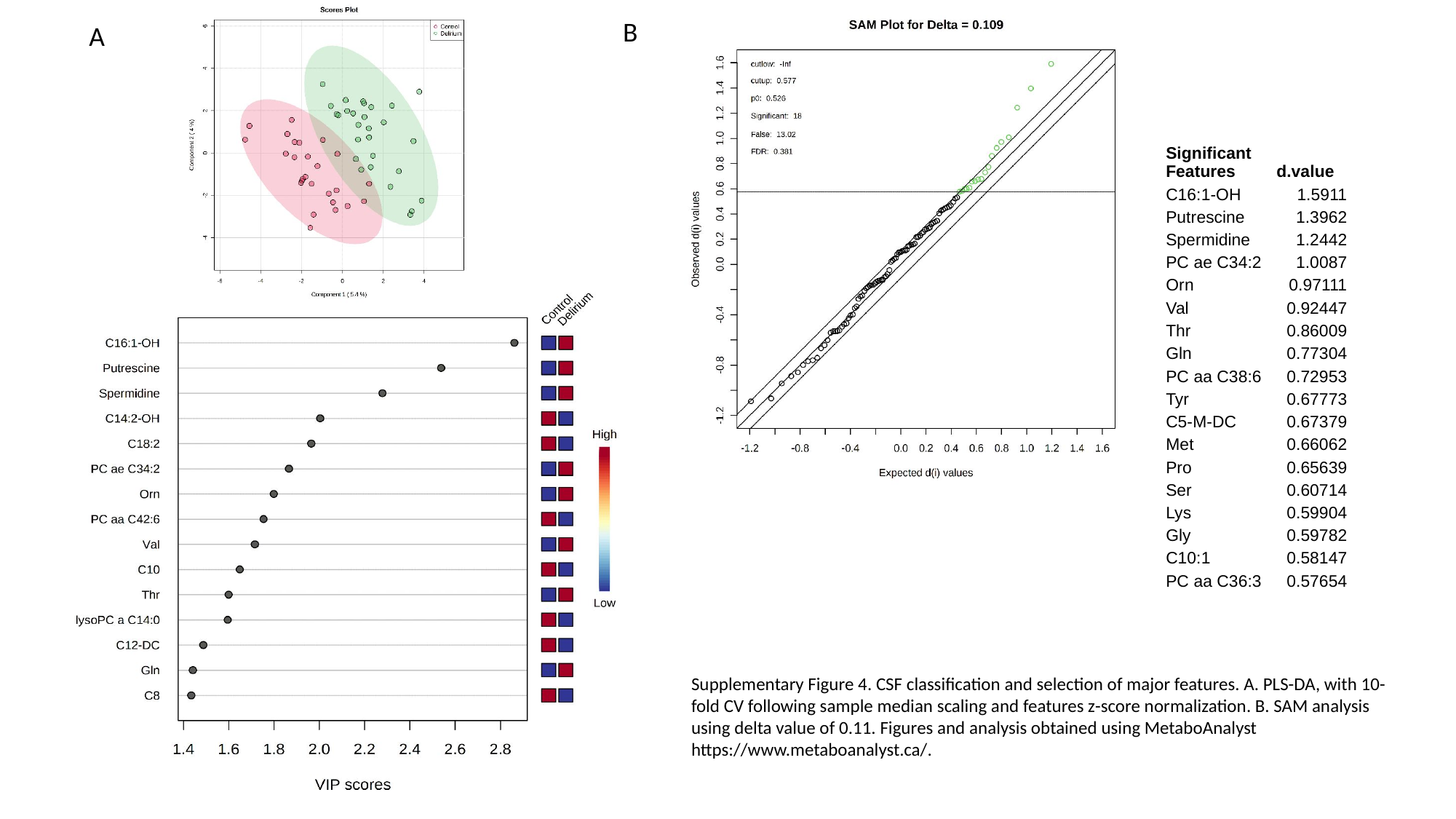

B
A
| Significant Features | d.value |
| --- | --- |
| C16:1-OH | 1.5911 |
| Putrescine | 1.3962 |
| Spermidine | 1.2442 |
| PC ae C34:2 | 1.0087 |
| Orn | 0.97111 |
| Val | 0.92447 |
| Thr | 0.86009 |
| Gln | 0.77304 |
| PC aa C38:6 | 0.72953 |
| Tyr | 0.67773 |
| C5-M-DC | 0.67379 |
| Met | 0.66062 |
| Pro | 0.65639 |
| Ser | 0.60714 |
| Lys | 0.59904 |
| Gly | 0.59782 |
| C10:1 | 0.58147 |
| PC aa C36:3 | 0.57654 |
Supplementary Figure 4. CSF classification and selection of major features. A. PLS-DA, with 10-fold CV following sample median scaling and features z-score normalization. B. SAM analysis using delta value of 0.11. Figures and analysis obtained using MetaboAnalyst https://www.metaboanalyst.ca/.

## Slide 5
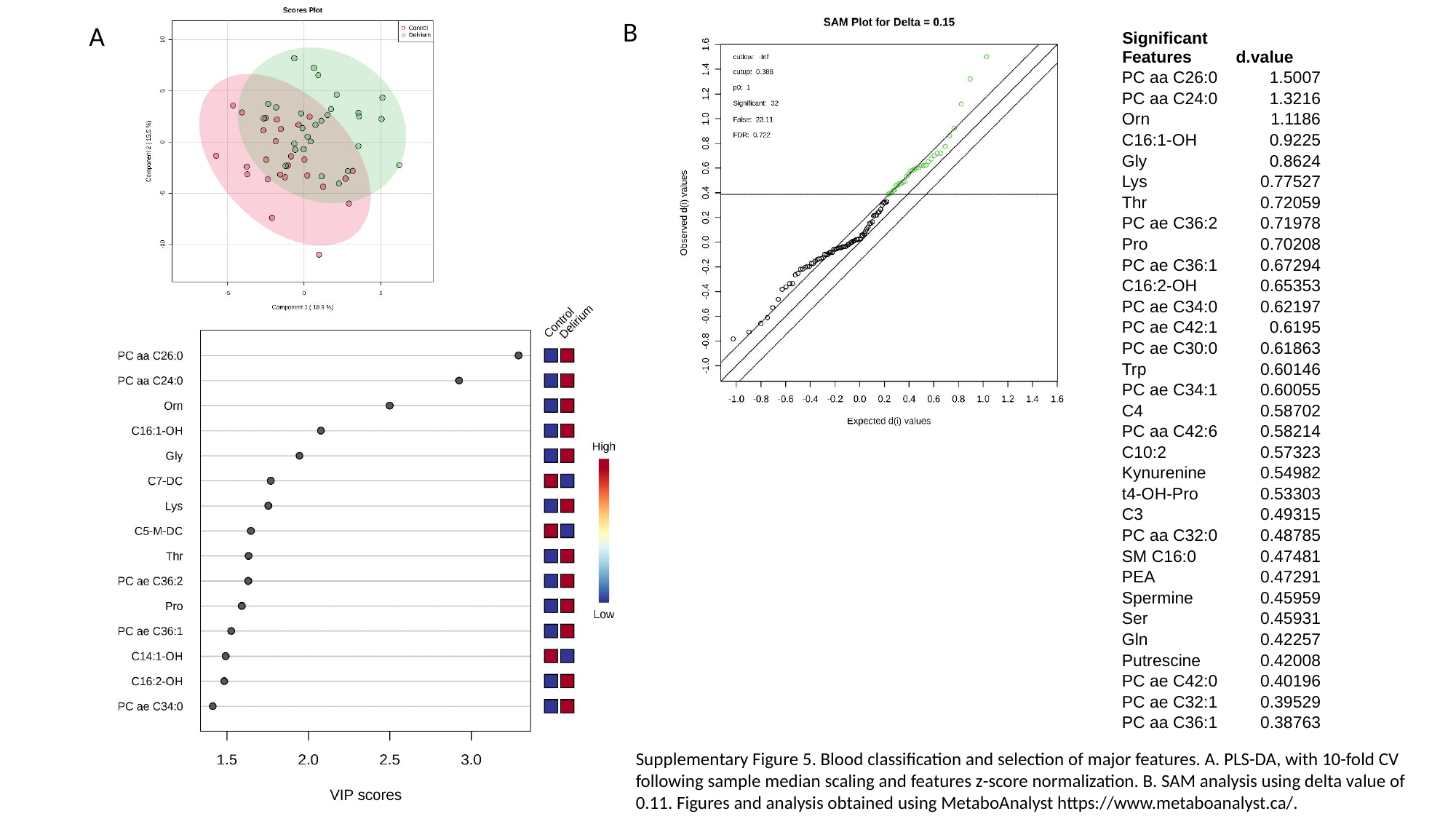

| Significant Features | d.value |
| --- | --- |
| PC aa C26:0 | 1.5007 |
| PC aa C24:0 | 1.3216 |
| Orn | 1.1186 |
| C16:1-OH | 0.9225 |
| Gly | 0.8624 |
| Lys | 0.77527 |
| Thr | 0.72059 |
| PC ae C36:2 | 0.71978 |
| Pro | 0.70208 |
| PC ae C36:1 | 0.67294 |
| C16:2-OH | 0.65353 |
| PC ae C34:0 | 0.62197 |
| PC ae C42:1 | 0.6195 |
| PC ae C30:0 | 0.61863 |
| Trp | 0.60146 |
| PC ae C34:1 | 0.60055 |
| C4 | 0.58702 |
| PC aa C42:6 | 0.58214 |
| C10:2 | 0.57323 |
| Kynurenine | 0.54982 |
| t4-OH-Pro | 0.53303 |
| C3 | 0.49315 |
| PC aa C32:0 | 0.48785 |
| SM C16:0 | 0.47481 |
| PEA | 0.47291 |
| Spermine | 0.45959 |
| Ser | 0.45931 |
| Gln | 0.42257 |
| Putrescine | 0.42008 |
| PC ae C42:0 | 0.40196 |
| PC ae C32:1 | 0.39529 |
| PC aa C36:1 | 0.38763 |
B
A
Supplementary Figure 5. Blood classification and selection of major features. A. PLS-DA, with 10-fold CV following sample median scaling and features z-score normalization. B. SAM analysis using delta value of 0.11. Figures and analysis obtained using MetaboAnalyst https://www.metaboanalyst.ca/.

## Slide 6
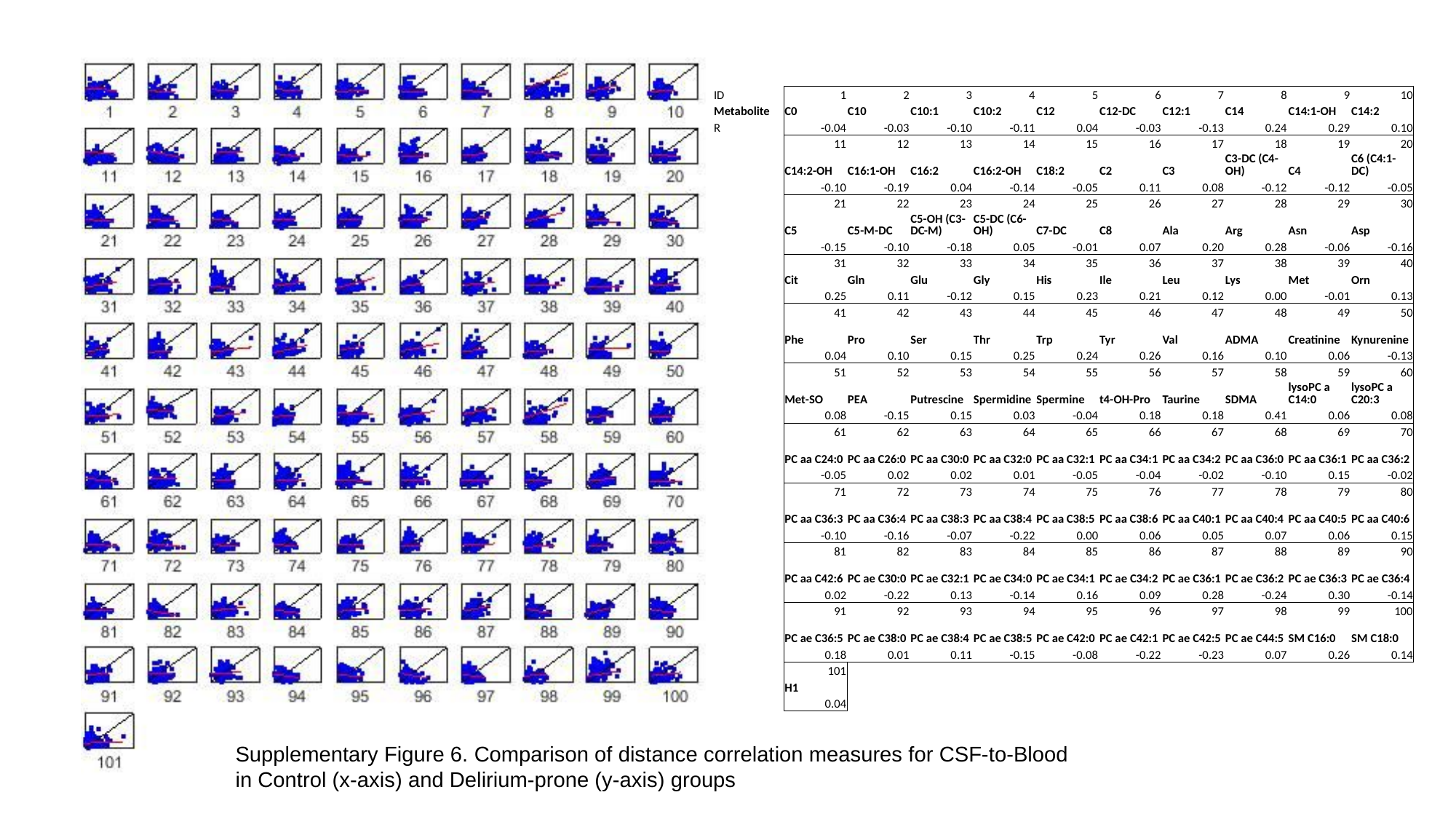

| ID | 1 | 2 | 3 | 4 | 5 | 6 | 7 | 8 | 9 | 10 |
| --- | --- | --- | --- | --- | --- | --- | --- | --- | --- | --- |
| Metabolite | C0 | C10 | C10:1 | C10:2 | C12 | C12-DC | C12:1 | C14 | C14:1-OH | C14:2 |
| R | -0.04 | -0.03 | -0.10 | -0.11 | 0.04 | -0.03 | -0.13 | 0.24 | 0.29 | 0.10 |
| | 11 | 12 | 13 | 14 | 15 | 16 | 17 | 18 | 19 | 20 |
| | C14:2-OH | C16:1-OH | C16:2 | C16:2-OH | C18:2 | C2 | C3 | C3-DC (C4-OH) | C4 | C6 (C4:1-DC) |
| | -0.10 | -0.19 | 0.04 | -0.14 | -0.05 | 0.11 | 0.08 | -0.12 | -0.12 | -0.05 |
| | 21 | 22 | 23 | 24 | 25 | 26 | 27 | 28 | 29 | 30 |
| | C5 | C5-M-DC | C5-OH (C3-DC-M) | C5-DC (C6-OH) | C7-DC | C8 | Ala | Arg | Asn | Asp |
| | -0.15 | -0.10 | -0.18 | 0.05 | -0.01 | 0.07 | 0.20 | 0.28 | -0.06 | -0.16 |
| | 31 | 32 | 33 | 34 | 35 | 36 | 37 | 38 | 39 | 40 |
| | Cit | Gln | Glu | Gly | His | Ile | Leu | Lys | Met | Orn |
| | 0.25 | 0.11 | -0.12 | 0.15 | 0.23 | 0.21 | 0.12 | 0.00 | -0.01 | 0.13 |
| | 41 | 42 | 43 | 44 | 45 | 46 | 47 | 48 | 49 | 50 |
| | Phe | Pro | Ser | Thr | Trp | Tyr | Val | ADMA | Creatinine | Kynurenine |
| | 0.04 | 0.10 | 0.15 | 0.25 | 0.24 | 0.26 | 0.16 | 0.10 | 0.06 | -0.13 |
| | 51 | 52 | 53 | 54 | 55 | 56 | 57 | 58 | 59 | 60 |
| | Met-SO | PEA | Putrescine | Spermidine | Spermine | t4-OH-Pro | Taurine | SDMA | lysoPC a C14:0 | lysoPC a C20:3 |
| | 0.08 | -0.15 | 0.15 | 0.03 | -0.04 | 0.18 | 0.18 | 0.41 | 0.06 | 0.08 |
| | 61 | 62 | 63 | 64 | 65 | 66 | 67 | 68 | 69 | 70 |
| | PC aa C24:0 | PC aa C26:0 | PC aa C30:0 | PC aa C32:0 | PC aa C32:1 | PC aa C34:1 | PC aa C34:2 | PC aa C36:0 | PC aa C36:1 | PC aa C36:2 |
| | -0.05 | 0.02 | 0.02 | 0.01 | -0.05 | -0.04 | -0.02 | -0.10 | 0.15 | -0.02 |
| | 71 | 72 | 73 | 74 | 75 | 76 | 77 | 78 | 79 | 80 |
| | PC aa C36:3 | PC aa C36:4 | PC aa C38:3 | PC aa C38:4 | PC aa C38:5 | PC aa C38:6 | PC aa C40:1 | PC aa C40:4 | PC aa C40:5 | PC aa C40:6 |
| | -0.10 | -0.16 | -0.07 | -0.22 | 0.00 | 0.06 | 0.05 | 0.07 | 0.06 | 0.15 |
| | 81 | 82 | 83 | 84 | 85 | 86 | 87 | 88 | 89 | 90 |
| | PC aa C42:6 | PC ae C30:0 | PC ae C32:1 | PC ae C34:0 | PC ae C34:1 | PC ae C34:2 | PC ae C36:1 | PC ae C36:2 | PC ae C36:3 | PC ae C36:4 |
| | 0.02 | -0.22 | 0.13 | -0.14 | 0.16 | 0.09 | 0.28 | -0.24 | 0.30 | -0.14 |
| | 91 | 92 | 93 | 94 | 95 | 96 | 97 | 98 | 99 | 100 |
| | PC ae C36:5 | PC ae C38:0 | PC ae C38:4 | PC ae C38:5 | PC ae C42:0 | PC ae C42:1 | PC ae C42:5 | PC ae C44:5 | SM C16:0 | SM C18:0 |
| | 0.18 | 0.01 | 0.11 | -0.15 | -0.08 | -0.22 | -0.23 | 0.07 | 0.26 | 0.14 |
| | 101 | | | | | | | | | |
| | H1 | | | | | | | | | |
| | 0.04 | | | | | | | | | |
Supplementary Figure 6. Comparison of distance correlation measures for CSF-to-Blood in Control (x-axis) and Delirium-prone (y-axis) groups

## Slide 7
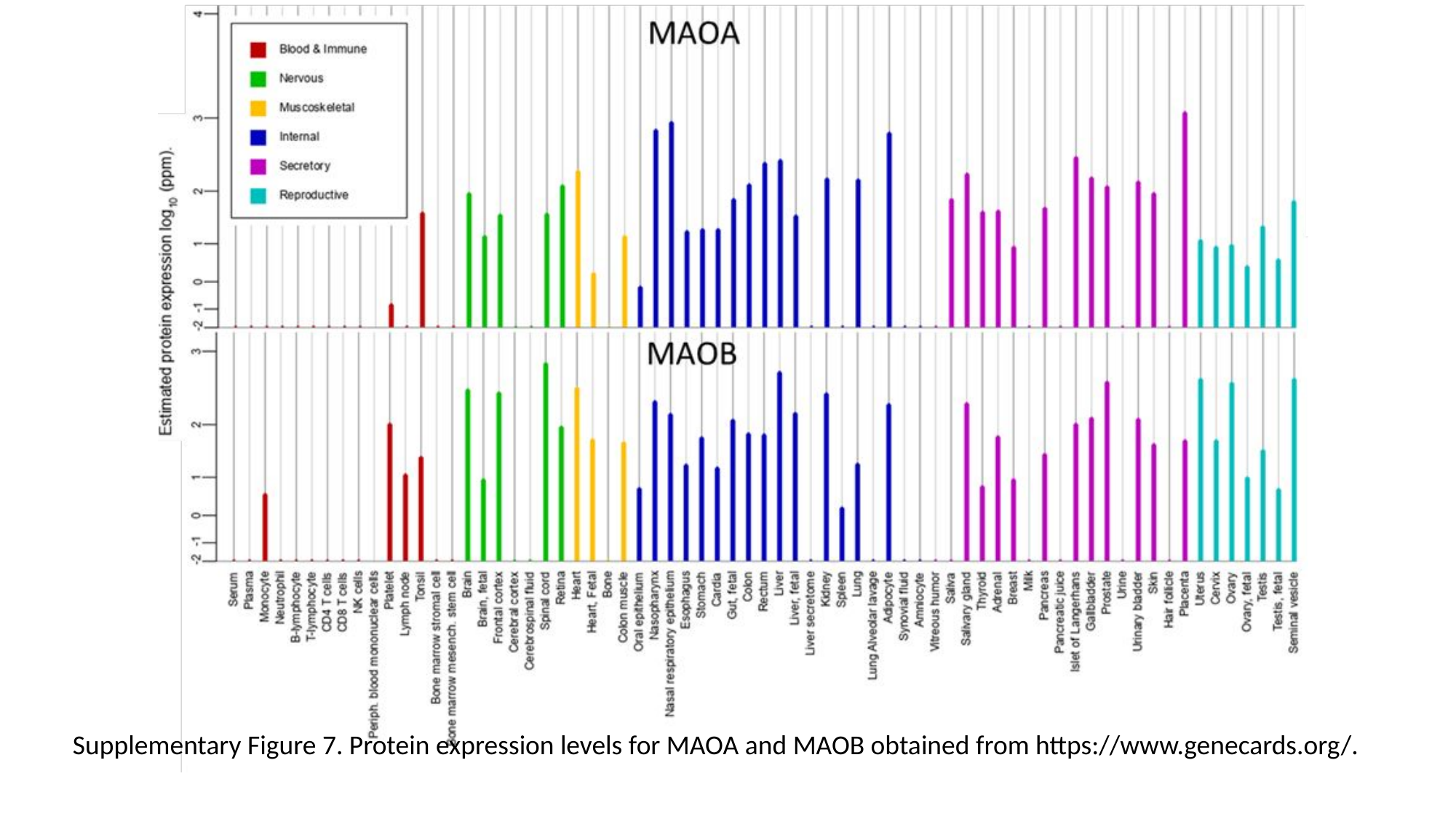

Supplementary Figure 7. Protein expression levels for MAOA and MAOB obtained from https://www.genecards.org/.

## Slide 8
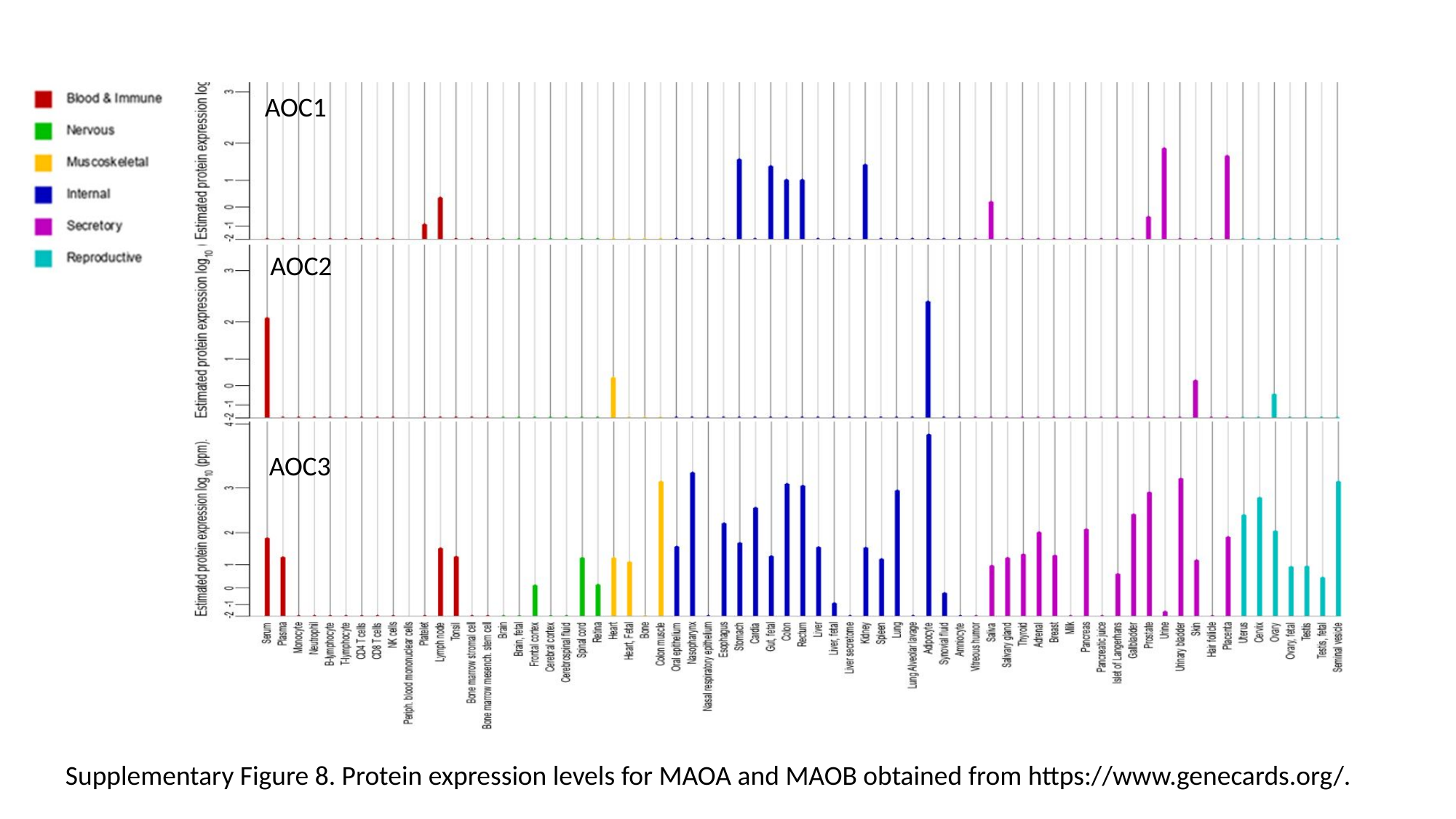

AOC1
AOC2
AOC3
Supplementary Figure 8. Protein expression levels for MAOA and MAOB obtained from https://www.genecards.org/.

## Slide 9
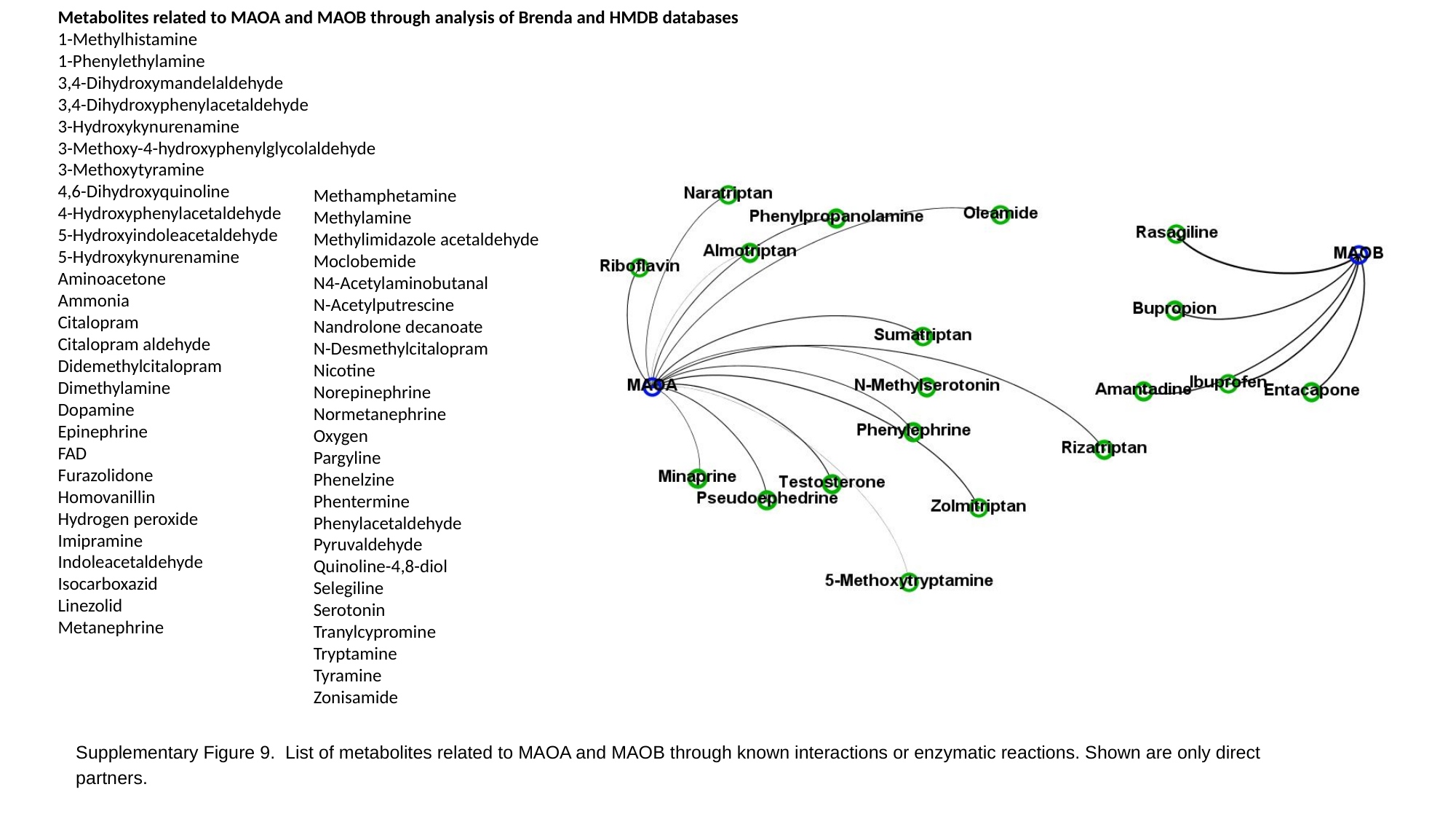

Metabolites related to MAOA and MAOB through analysis of Brenda and HMDB databases
1-Methylhistamine
1-Phenylethylamine
3,4-Dihydroxymandelaldehyde
3,4-Dihydroxyphenylacetaldehyde
3-Hydroxykynurenamine
3-Methoxy-4-hydroxyphenylglycolaldehyde
3-Methoxytyramine
4,6-Dihydroxyquinoline
4-Hydroxyphenylacetaldehyde
5-Hydroxyindoleacetaldehyde
5-Hydroxykynurenamine
Aminoacetone
Ammonia
Citalopram
Citalopram aldehyde
Didemethylcitalopram
Dimethylamine
Dopamine
Epinephrine
FAD
Furazolidone
Homovanillin
Hydrogen peroxide
Imipramine
Indoleacetaldehyde
Isocarboxazid
Linezolid
Metanephrine
Methamphetamine
Methylamine
Methylimidazole acetaldehyde
Moclobemide
N4-Acetylaminobutanal
N-Acetylputrescine
Nandrolone decanoate
N-Desmethylcitalopram
Nicotine
Norepinephrine
Normetanephrine
Oxygen
Pargyline
Phenelzine
Phentermine
Phenylacetaldehyde
Pyruvaldehyde
Quinoline-4,8-diol
Selegiline
Serotonin
Tranylcypromine
Tryptamine
Tyramine
Zonisamide
Supplementary Figure 9. List of metabolites related to MAOA and MAOB through known interactions or enzymatic reactions. Shown are only direct partners.

## Slide 10
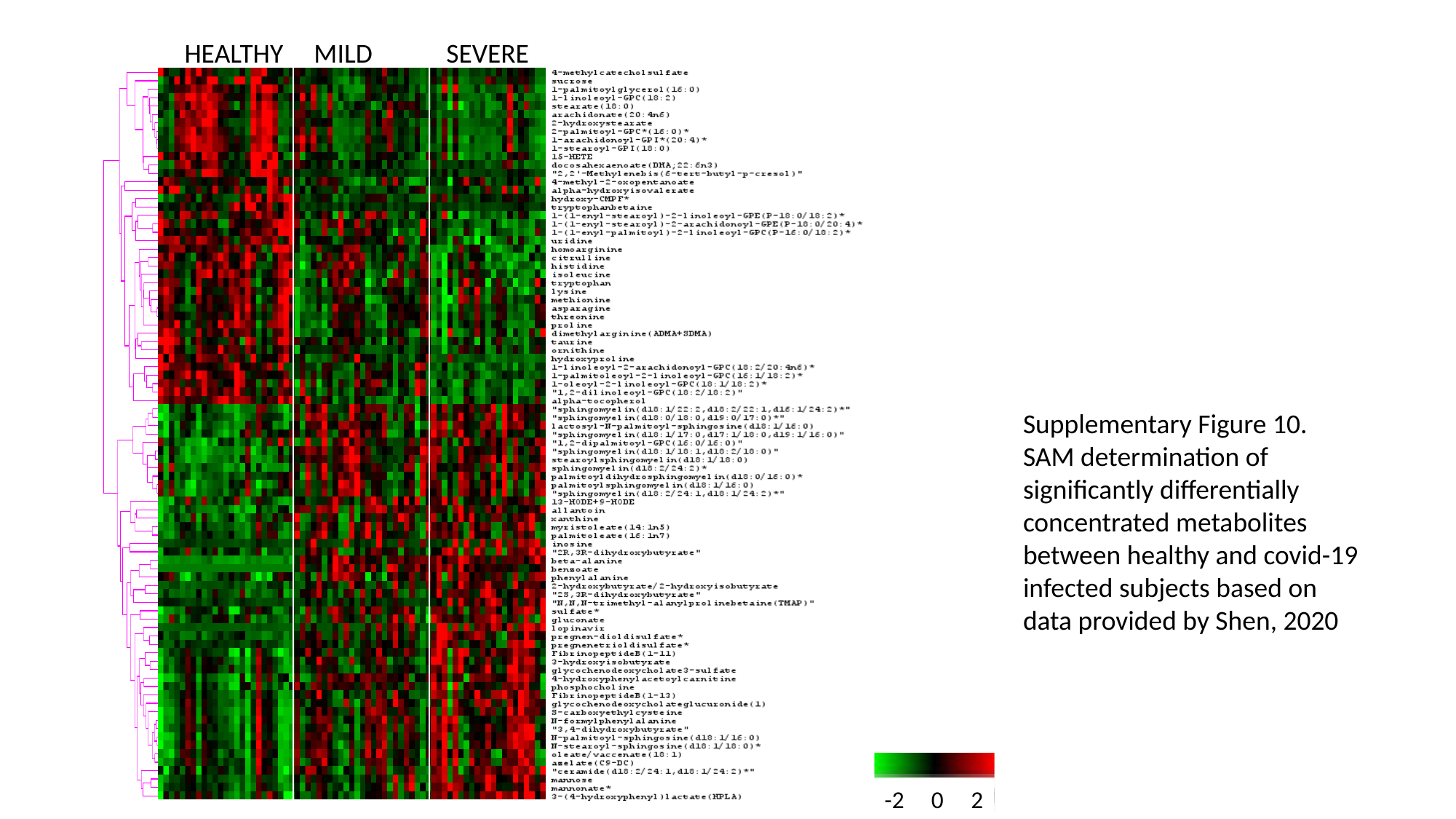

HEALTHY MILD SEVERE
Supplementary Figure 10.
SAM determination of significantly differentially concentrated metabolites between healthy and covid-19 infected subjects based on data provided by Shen, 2020
-2 0 2

## Slide 11
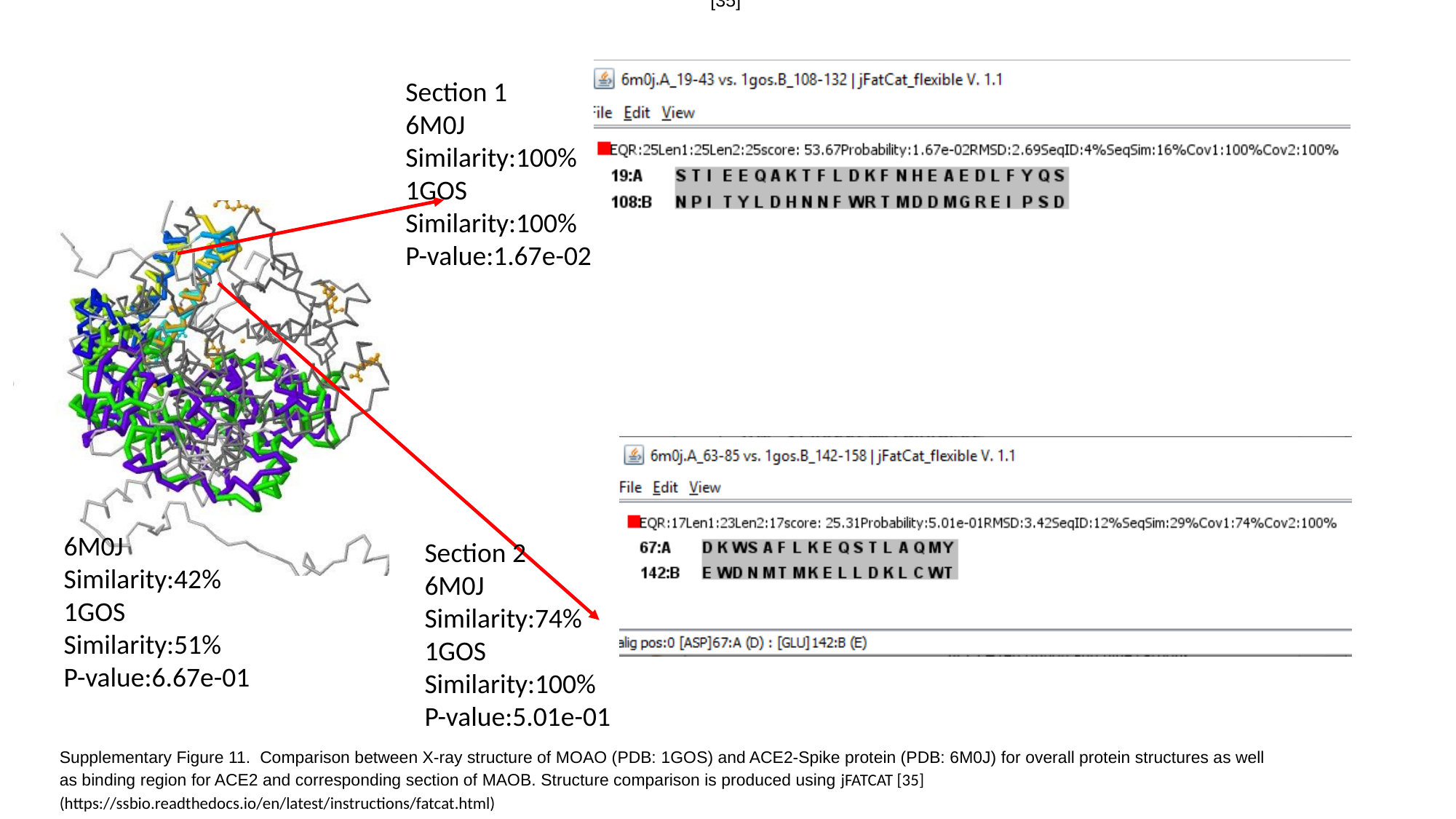

[35]
Section 1
6M0J
Similarity:100%
1GOS
Similarity:100%
P-value:1.67e-02
6M0J
Similarity:42%
1GOS
Similarity:51%
P-value:6.67e-01
Section 2
6M0J
Similarity:74%
1GOS
Similarity:100%
P-value:5.01e-01
Supplementary Figure 11. Comparison between X-ray structure of MOAO (PDB: 1GOS) and ACE2-Spike protein (PDB: 6M0J) for overall protein structures as well as binding region for ACE2 and corresponding section of MAOB. Structure comparison is produced using jFATCAT [35] (https://ssbio.readthedocs.io/en/latest/instructions/fatcat.html)
